# Tackling exosome and nuclear receptor interaction: an emerging paradigm in the treatment of chronic diseases

**DOI:** 10.1186/s40779-024-00564-1

**Published:** 2024-09-26

**Authors:** Babu Santha Aswani, Mangala Hegde, Ravichandran Vishwa, Mohammed S. Alqahtani, Mohamed Abbas, Hassan Ali Almubarak, Gautam Sethi, Ajaikumar B. Kunnumakkara

**Affiliations:** 1 Cancer Biology Laboratory, Department of Biosciences and Bioengineering, Indian Institute of Technology Guwahati (IITG), Guwahati, 781039 Assam India; 2 Radiological Sciences Department, College of Applied Medical Sciences, King Khalid University, 61421 Abha, Saudi Arabia; 3 BioImaging Unit, Space Research Centre, Michael Atiyah Building, University of Leicester, Leicester, LE1 7RH UK; 4 Electrical Engineering Department, College of Engineering, King Khalid University, 61421 Abha, Saudi Arabia; 5 Division of Radiology, Department of Medicine, College of Medicine and Surgery, King Khalid University, 61421 Abha, Saudi Arabia; 6 Department of Pharmacology, Yong Loo Lin School of Medicine, National University of Singapore, Singapore, 117600 Singapore; 7 NUS Centre for Cancer Research, Yong Loo Lin School of Medicine, National University of Singapore, Singapore, 117699 Singapore

**Keywords:** Nuclear receptors, Exosomes, Chronic diseases, Inflammation, MicroRNAs

## Abstract

Nuclear receptors (NRs) function as crucial transcription factors in orchestrating essential functions within the realms of development, host defense, and homeostasis of body. NRs have garnered increased attention due to their potential as therapeutic targets, with drugs directed at NRs demonstrating significant efficacy in impeding chronic disease progression. Consequently, these pharmacological agents hold promise for the treatment and management of various diseases. Accumulating evidence emphasizes the regulatory role of exosome-derived microRNAs (miRNAs) in chronic inflammation, disease progression, and therapy resistance, primarily by modulating transcription factors, particularly NRs. By exploiting inflammatory pathways such as protein kinase B (Akt)/mammalian target of rapamycin (mTOR), nuclear factor kappa-B (NF-κB), signal transducer and activator of transcription 3 (STAT3), and Wnt/β-catenin signaling, exosomes and NRs play a pivotal role in the panorama of development, physiology, and pathology. The internalization of exosomes modulates NRs and initiates diverse autocrine or paracrine signaling cascades, influencing various processes in recipient cells such as survival, proliferation, differentiation, metabolism, and cellular defense mechanisms. This comprehensive review meticulously examines the involvement of exosome-mediated NR regulation in the pathogenesis of chronic ailments, including atherosclerosis, cancer, diabetes, liver diseases, and respiratory conditions. Additionally, it elucidates the molecular intricacies of exosome-mediated communication between host and recipient cells via NRs, leading to immunomodulation. Furthermore, it outlines the implications of exosome-modulated NR pathways in the prophylaxis of chronic inflammation, delineates current limitations, and provides insights into future perspectives. This review also presents existing evidence on the role of exosomes and their components in the emergence of therapeutic resistance.

## Background

Chronic diseases represent enduring non-communicable conditions that persist for over an year, significantly impacting daily life functions and necessitates medical care [1, 2]. Despite recent advancements in medicine contributing to increased life expectancy among affected individuals, chronic disease remains a predominant global challenge [3, 4]. The World Health Organization’s independent high-level commission on non-communicable diseases stated that cardiovascular diseases (CVDs), cancer, chronic respiratory diseases, and diabetes are the top 4 chronic diseases, collectively responsible for a substantial number of fatalities across all age demographics globally [5]. Importantly, the etiology and progression of these diseases are rooted in modifiable risk factors, including insufficient physical activity, suboptimal dietary practices, use of tobacco, alcohol consumption, stress, and radiation exposure [6, 7]. Moreover, cellular cross-talk plays a pivotal role in the pathogenesis of chronic diseases facilitated by proteins such as receptors, ligands, and transcription factors which actively engage in cellular communication processes [8–10]. Nuclear receptors (NRs) constitute one such family of ligand-activated transcription factors that are crucial in various biological processes such as development, reproduction, metabolism, and defense [11–15]. The human NR superfamily comprises 48 members characterized by conserved structures, including an α-helical globular region in the C-terminal for ligand binding and dimerization, a hinge region connecting the C-terminal to the deoxyribonucleic acid (DNA) binding region, and a variable N-terminal aiding in transcriptional regulation [16–19]. Recent studies have focused on targeting NRs to develop innovative treatment approaches for chronic diseases [20–25]. Specifically, the modulation of NRs through agonists, antagonists, or miRNAs induces transcriptional regulation of downstream genes that govern metabolic processes [26–28]. Importantly, exosomal contents such as miRNAs and long non-coding RNAs (lncRNAs) interact and modulate NRs resulting in perturbations in associated immune cells, inflammatory cytokines, reactive oxygen species (ROS), and cell cycle regulators [29–31]. Studies have also revealed that treatment with NR agonists induces the secretion of exosomes, thereby modulating cellular functions [32, 33].

Recently, extracellular vesicles (EVs) have garnered substantial attention as pivotal mediators of cellular signaling, emerging as both diagnostic and prognostic biomarkers in the context of chronic diseases, thus constituting a prominent subject of scientific inquiry [34–37]. EVs are membrane-bound microparticles that facilitate the transfer of molecular cargo from donor cells to recipient cells [38, 39]. Based on their size and origin, EVs are classified as apoptotic bodies, ectosomes, endosomes, exosomes, microparticles, microvesicles, nanoparticles, and oncosomes [40]. Exosomes are defined as EV falling within a defined size range of 30–150 nm [41, 42]. They originate from the inward budding of the early endosomal membrane, can be isolated through particular methodologies and contain specific cargo [43–45]. The role of exosomes in transporting biological contents, including lipids, proteins, and nucleic acids is noteworthy because they influence both physiological and pathological processes [43, 46–49]. These cargos are effectively delivered to recipient cells through the communication via surface proteins of exosomes, internalization through endocytosis, or direct fusion with recipient cells [41, 42]. A substantial body of research suggests the role of exosomes in the conversion of acute diseases into chronic conditions [50–54]. However, recent investigations have emphasized the therapeutic potential of exosomes, primarily attributed to their low immunogenicity, nanoscale size, targeted delivery of cargo, biocompatibility, and minimal toxicity [35, 55, 56].

**Fig. 1:**
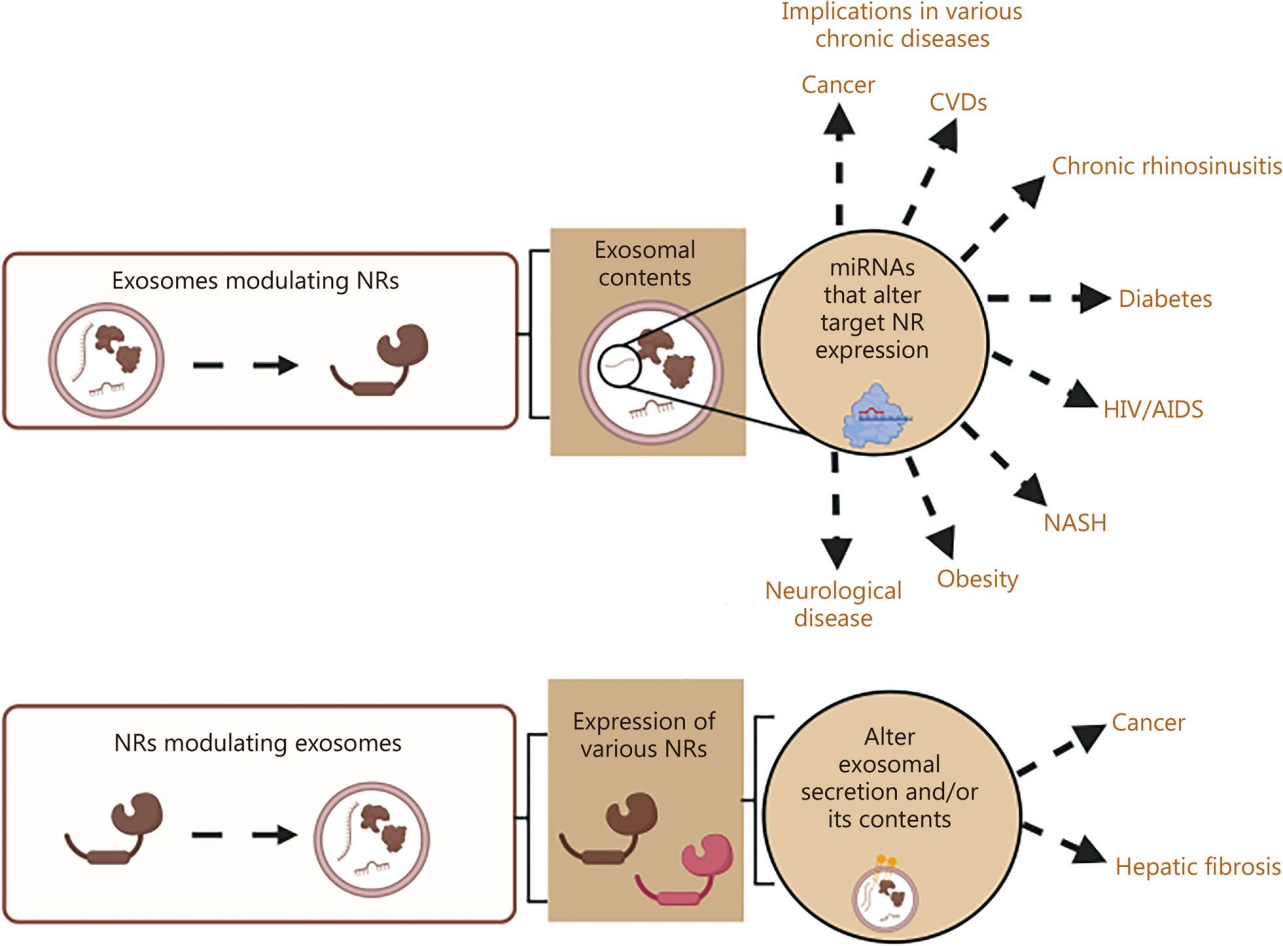
The interaction between exosomes and nuclear receptors (NRs) plays a crucial role in the development and progression of various chronic diseases. Exosomes can influence NRs by modulating their expression through the transfer of their contents, such as microRNAs (miRNAs). This alteration in NR expression affects downstream targets, contributing to the onset and progression of chronic diseases. Conversely, NRs can also regulate exosomes by modifying their secretion or altering their contents that are involved in patho-physiological conditions. AIDS acquired immune deficiency syndrome, CVDs cardiovascular diseases, HIV human immunodeficiency virus, NASH non-alcoholic steatohepatitis

Interestingly, recent studies highlight the significance of the intricate communication between exosomes and NRs in disease development and treatment [32, 33]. This has been summarized in Fig. 1. This review provides a comprehensive overview of the cross-talk between exosomes and NRs, elucidating the mechanisms involved in the development and prophylaxis of chronic diseases. Further, the review delves into the exosomal content, alterations in gene expression, and underlying pathways influencing the progression or regression of chronic diseases. Current limitations and future goals for developing treatment regimens against persistent chronic ailments that target exosomes and NR-mediated cross-talk were also discussed.

## NRs and signaling

NRs constitute a substantial group of ligand-dependent transcription factors that play a crucial role in cellular signaling and metabolism. As mentioned previously, this superfamily comprises 48 members, including steroid, thyroid, and vitamin receptors [13, 14, 57–63]. Further, they can be classified into three groups based on their ligands and modes of action. Class I includes the endocrine receptors, such as androgen receptor (AR), estrogen receptor alpha (ERα), ERβ, progesterone receptor (PR), retinoic acid receptor alpha (RARα), RARβ, RARγ, thyroid hormone receptor alpha (THRα), THRβ, vitamin D receptor (VDR), glucocorticoid receptor (GR), and mineralocorticoid receptor (MR); class II comprises the orphan receptors with no known ligands including chicken ovalbumin upstream promoter transcription factors, dosage-sensitive sex-reversal adrenal hypoplasia congenital critical region on the X chromosome gene 1 (DAX1), germ cell nuclear factor, liver receptor homologue 1 (LRH1), tailless, photoreceptor-specific nuclear receptor, small heterodimer partner, and testicular receptor (TR); and the class III encompasses adopted NRs which were previously considered orphans, whose ligands have been subsequently discovered including constitutive androstane receptor (CAR), estrogen-related receptor alpha (ERRα), ERRβ, ERRγ, hepatocyte nuclear factor 4 alpha (HNF4α), HNF4γ, farnesoid X receptor (FXR), liver X receptor alpha (LXRα), LXRβ, neuron-derived orphan receptor 1, nerve growth factor-induced clone B, Nur-related factor 1, peroxisome proliferator-activated receptor alpha (PPARα), PPARγ, PPARδ, pregnane X receptor (PXR), Rev-ErbAα, Rev-ErbAβ, RAR-related orphan receptor alpha (RORα), RORβ, RORγ, retinoid X receptor alpha (RXRα), RXRβ, RXRγ, steroidogenic factor-1, and TR4 [13, 60, 64–66].

NRs exhibit a variety of structural forms, including monomers, homodimers, and heterodimers, each of which binds to specific DNA sequences known as “hormone-response elements” with the consensus sequence RGGTCA (where R denotes a purine base) [67]. NRs are classified into 4 subtypes according to their downstream signaling mechanisms. Type I receptors (e.g., ARs, ERs, and PRs) are primarily located in the cytoplasm and are bound by chaperone proteins [67]. Upon binding to a ligand, they dissociate from chaperones, facilitating homodimerization, nuclear translocation, and subsequent DNA binding with coactivators. Conversely, type II receptors (e.g., THRs, RARs) mainly reside in the nucleus and are pre-associated with specific DNA response elements. They may also form heterodimers with RXRs [67]. Binding of ligand to the specific domain prompts corepressor dissociation and coactivator recruitment [67]. Type III receptors share similarities with type I receptors but differ in hormone response element organization, while type IV receptors preferentially bind as monomers to half-site hormone-response elements [67].

It is increasingly acknowledged that NRs not only regulate the expression of target genes but also interact with other signaling pathways and their downstream effectors, thereby influencing each other’s activities [68]. This interplay reflects the integration of NR function within the cellular context. For example, NRs can reciprocally repress activator protein 1 (AP1) (c-Fos/c-Jun) activities, cross-talk with NF-κB pathway, and  be modulated through phosphorylation by the mitogen activated protein kinase (MAPK) pathway [19, 68, 69].

NRs act as substrates for various kinases that are activated by diverse signaling pathways. Phosphorylation of the N-terminal A/B region of NRs such as ERα, ERβ, PPARα, and AR by extracellular signal-regulated kinase (ERK), p38 MAPK, c-Jun NH2-terminal kinase (JNKs), and Akt promotes coactivator recruitment. This phosphorylation enhances chromatin remodeling, thereby increasing transcriptional efficiency and target gene expression [70–74]. Additionally, phosphorylation augments the growth-stimulating effects of certain NRs, including ERα and AR [72]. Src kinases phosphorylate ER at tyrosine 537 and protein kinase A (PKA) phosphorylates RARα at serine 369, positively modulating their transcriptional activities [75–77]. Cross-talk between NRs and signaling pathways also induces phosphorylation of NR coregulators, such as steroid receptor coactivator 1, PPARγ coactivator-1 alpha (PGC-1α), nuclear receptor coactivator 2 (NCOA2/TIF2), p300/CREB binding protein (CBP), and nuclear receptor coactivator 3 (NCOA3/pCIP), enhancing ligand binding efficacy and histone acetyltransferase recruitment [78–83]. Conversely, phosphorylation can deactivate NRs; for instance, protein kinase C (PKC) induced phosphorylation of VDR amino acid residues involved in response elements leads to suppression of gene expression [72, 84]. Phosphorylation-mediated inhibition of ERα and RARα activities occurs through the phosphorylation of residues within the DNA binding domain dimerization surface by PKA or PKC, respectively [72, 85, 86].

Aberrant phosphorylation of NRs is a crucial factor in the development and progression of various cancers, including breast, ovarian, and prostate cancers. The activation of MAPK and Akt kinases in tumors contributes to ligand-independent transactivation of ERs and ARs, leading to hormone-independent growth and resistance to hormone-based therapies such as androgen ablation or tamoxifen treatment in cancer cells [77, 87, 88].

## NRs and chronic diseases

**Fig. 2:**
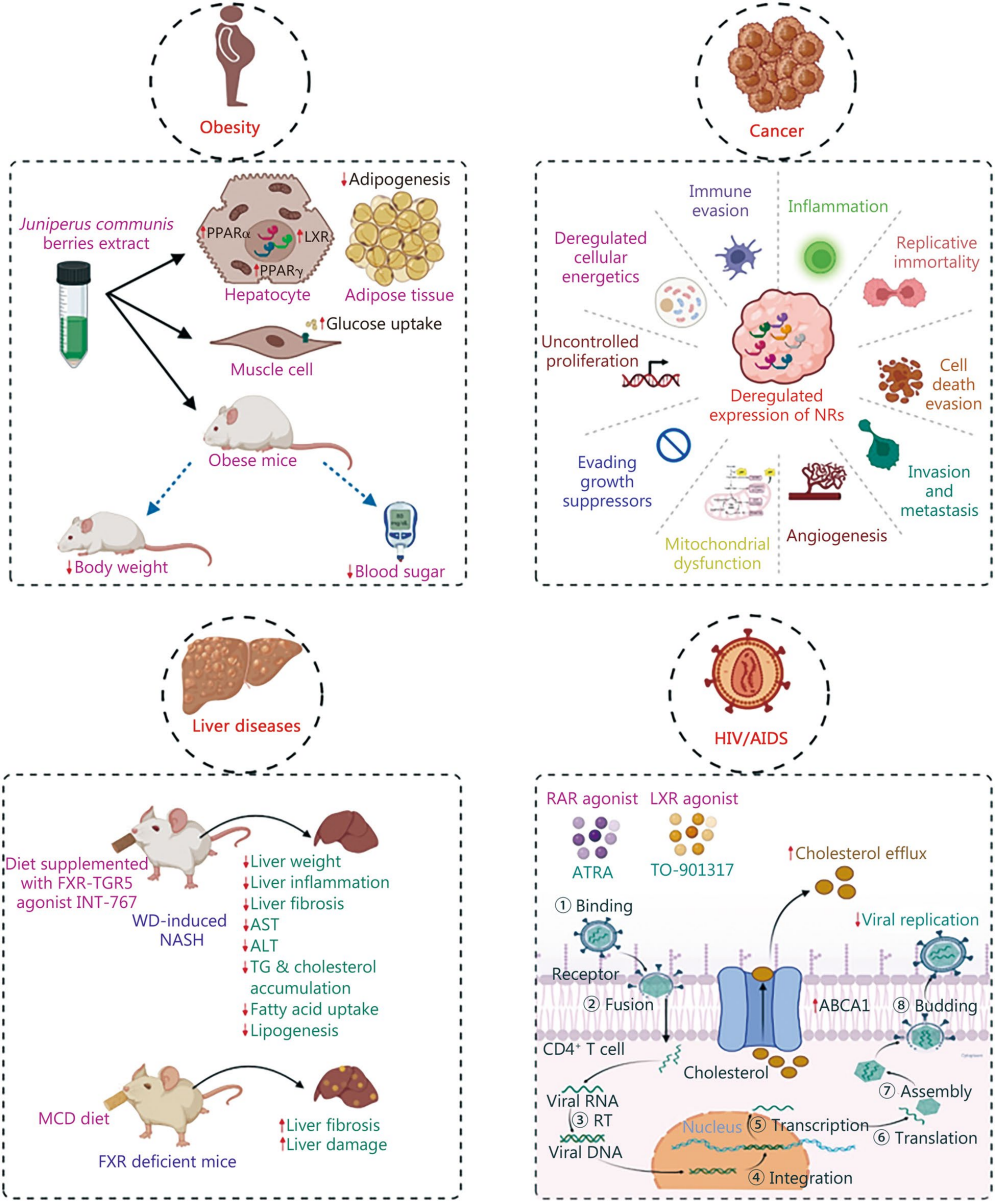
The pivotal role of nuclear receptors (NRs) in the etiology of chronic diseases. Aberrations in NR function are implicated in the initiation and progression of pathological states. NRs are crucial participants in the genesis and advancement of obesity, a condition primarily arising from lifestyle factors and associated inflammatory processes. Targeting NRs represents a significant avenue for obesity management. Administration of a methanol extract derived from *Juniperus communis* berries led to a reduction in weight and blood sugar levels in obese mice by upregulating PPARα, PPARγ and LXR. Moreover, deregulated expression of NRs has been implicated in the manifestation of various cancer hallmarks, including immune evasion, inflammation, replicative immortality, cell death evasion, invasion and metastasis, angiogenesis, mitochondrial dysfunction, evading growth suppressors, uncontrolled proliferation, and deregulated cellular energetics. Of note, the dual agonist of FXR and TGR5, INT-767, has exhibited therapeutic efficacy in countering non-alcoholic steatohepatitis (NASH) and combinatorial treatment involving RAR and LXR agonists, namely all-trans retinoic acid (ATRA) and TO-901317, respectively, has resulted in diminished replication of the HIV. AIDS acquired immune deficiency syndrome, HIV human immunodeficiency virus, PPAR peroxisome proliferator-activated receptor, LXR liver X receptor, ALT alanine transaminase, AST aspartate aminotransferase, MCD methionine-choline deficient, ABCA1 ATP-binding cassette A1, RAR retinoic acid receptor, TG triglycerides, RT reverse transcriptase

Numerous investigations have consistently reported a correlation between metabolic reprogramming and the onset and progression of chronic diseases [89–91]. At cellular level, transcription factors exhibit the capacity to discern changes in metabolite levels and then modulate genes involved in diverse metabolic pathways such as glucose metabolism, lipid metabolism, insulin signaling cascade, and amino acid metabolism [92–100]. Consequently, disruption in genetics and alterations in their expression patterns result in metabolic dysregulation and pathological conditions [101–105]. Enhanced activity of certain cytoplasmic transcription factors, including signal transducers and activators of transcription (STAT), NF-κB, β-catenin, notch intracellular domain (NICD), AP1, hypoxia-inducible factors (HIF), myelocytomatosis oncogene (Myc), retinoblastoma binding protein (E2F), E26 transformation-specific (ETS) transcription factor and NRs has been observed in human cancers [106, 107]. The adaptability of NRs to promptly and dynamically respond to environmental stimuli makes them versatile integral components of cells. A multitude of NR types are expressed in different tissues throughout the body, exhibiting responsiveness to a wide range of steroids, non-steroidal hormones, metabolites, and molecular signals including phosphorylation and acetylation [108]. Consequently, the orchestrated activities of NRs hold significance in both physiological processes and pathological conditions. Within this context, NRs have undergone exquisite evolutionary refinement to regulate diverse fuel sources, including dietary and endogenous fat (PPAR), cholesterol (LXR; FXR), sugar mobilization (GR), salt (MR), and calcium (VDR) [109]. The THR evolved to maintain basal metabolic rate, while reproductive processes are controlled by gonadal steroid receptors (PR, ER, AR). Additionally, the NR superfamily manages inflammation during infection by defending the body while suppressing appetite and promoting sleep [109]. An ill body can defend itself by mobilizing fuel reserves, transiently suppressing inflammation, and releasing adrenal steroids. Clinically, glucocorticoids are mainly used as anti-inflammatory agents. Receptors like RARs, LXRs, PPARγ, PPARδ, and VDR protect against inflammation, revealing the dual role of the NR superfamily in governing energy homeostasis and the inflammatory response [109]. Moreover, the xenobiotic receptors such as PXR and CAR have evolved to counteract myriads of environmental toxins [109]. In summary, nature has sculpted within this superfamily of receptors a cohesive ability to govern both energy homeostasis and the inflammatory response, highlighting the inherent duality between these physiological systems. Therefore, dysfunction in NR signaling  can leads to proliferative, metabolic and reproductive-related chronic diseases such as cancer, obesity, diabetes, and infertility. This has been summarized in Fig. 2 [110–113].

Pioneering Nobel Prize-winning research initially established a connection between steroid hormones and prostate cancer, and later expanded this association to breast cancer [114, 115]. Subsequent investigations have firmly established the significant contribution of NR signaling to the development and progression of cancer. This is supported by the frequent dependence of oncogenic events leading to cell transformation on cascades of NR-mediated transcriptional signaling, as well as the reported aberrant expression of NRs in several types of cancer [66]. For instance, overexpression of ER occurs in more than 70% of breast cancers, making it as a key therapeutic target [116]. PR has been shown to enhance motility and invasiveness in breast cancer, while AR activation by androgens is crucial for the initiation and progression of prostate cancers [117, 118]. RAR activated by retinoic acid, exerts antiproliferative effects in tumor cells and translocation or fusion involving promyelocytic leukemia with RARα leads to acute promyelocytic leukemia (APL) in hematopoietic myeloid cells [119]. Early studies have highlighted the crucial role of NR status in cancer, particularly in terms of patient survival and treatment outcomes. For example, tumors lacking expression of both ER and PR (ER^−^PR^−^) exhibit resistance to hormone therapies and present the least favorable prognosis for patient survival. Subsequently, tumors characterized by the absence of ER but the presence of PR (ER^−^PR^+^) have an intermediate prognosis. In contrast, tumors expressing both ER and PR (ER^+^PR^+^) generally demonstrate more favorable prognoses [120–122]. Additionally, the status of ER and PR along with HER2 has established a straightforward stratification for breast cancer which correlates with both survival outcomes and the selection of targeted therapeutic approaches [120, 121]. Another study has demonstrated the overexpression of LRH1 in breast cancer cells playing pivotal role in inducing proliferation, invasion and migration in both ER^+^ and ER^−^ breast cancer cells [123]. Additionally, recent studies have also clarified that HNF4α plays a central role in the oncogenesis of colorectal cancers through the regulation of ROS generation [124, 125]. To date, the most successful therapeutic targeting transcription factors in cancer has predominantly stemmed from the utilization of small molecules designed to selectively bind to nuclear hormone receptors [126]. Notably, pharmaceutical agents modulating the activity of ER, AR, RAR, and GR are presently employed in the treatment regimens for breast cancer, prostate cancer, APL, and acute lymphoblastic leukemia, respectively [18, 126].

A multitude of studies have elucidated the essential role played by various NRs in the initiation and progression of other chronic diseases. Wang et al. [111] investigated the therapeutic potential of the FXR and G protein-coupled receptor 5 (TGR5) axis in the treatment of non-alcoholic steatohepatitis (NASH) [111]. Administration of FXR-TGR5 dual agonist, INT-767, effectively impeded the advancement of hepatic inflammation, steatosis, and fibrosis in C57BL/6J mice subjected to a Western diet. Notably, INT-767 also exhibited inhibitory effects on fatty acid synthesis and uptake, cholesterol uptake, and bile acid hydrophobicity. Mechanistic investigations revealed that INT-767 upregulated the expression of pAMP-activated protein kinase, sirtuin (SIRT)-1, SIRT-3, and PGC1α in the liver, further elucidating its molecular mechanisms [111]. Another study showed the importance of the FXR in the pathogenesis of non-alcoholic fatty liver disease (NAFLD) in murine models. When subjected to a methionine-choline deficient (MCD) diet, FXR-deficient mice exhibited increased hepatic fibrosis and liver damage but reduced steatosis compared to wild-type counterparts fed with MCD [112]. Additionally, the FXR-deficient mice inhibited the expression of genes involved in fatty acid uptake and triglyceride accumulation [112]. Furthermore, an in vitro study highlighted the beneficial effects of all-trans-retinoic acid (ATRA), a ligand for RAR, in ameliorating podocyte injury [127]. ATRA treatment activated podocin and nephrin while inhibited transforming growth factor beta (TGF-β)1 in murine podocytes, emphasizing the involvement of RAR pathway in ATRA-induced differentiation of injured podocytes [127]. Moreover, treatment with *Juniperus communis* berries showed activation of PPARα, PPARγ, and LXR, while this treatment led to reduced body weight and fasting glucose levels in high-fat diet (HFD) fed mice [110]. This study demonstrated the crucial role of NRs in cellular metabolism and their potential as targets for addressing obesity and diabetes [110]. Lastly, the study by Jiang et al. [113] highlighted the synergistic effect of RAR and LXR agonists, ATRA or TO-901317, in the context of human immunodeficiency virus (HIV) infection. Treatment with these agonists in CD4^+^ T cells was shown to enhance cholesterol efflux, thereby reducing intracellular cholesterol levels and preventing HIV entry into the cell [113]. These findings collectively indicate the diverse and crucial roles of NRs in various physiological and pathological processes, thereby substantiating their potential as targets for therapeutic interventions.

The chemistry of NRs, their ligand binding properties, and wide range of physiological functions have made them successful therapeutic targets [126]. Pharmaceutical agents that act as agonists or antagonists for NRs, such as tamoxifen for ER (used in breast cancer treatment), glitazones and thiazolidinediones for PPARγ (utilized in type II diabetes management), or dexamethasone for GR (applied in the treatment of inflammatory diseases), represent prevalent and widely employed therapeutic modalities [126, 128].

The combination of these investigations collectively suggests that NRs play a pivotal role in processes such as sugar mobilization, salt balance, calcium balance, fatty acid uptake, metabolism, cholesterol influx/efflux, and fat distribution. Experimental models with genetic deficiencies in NRs have exhibited disruptions in lipid metabolism, causing harm to host tissues. Therefore, NRs emerge as crucial mediators in the regulation of lipid metabolism, and any modulation thereof is anticipated to give rise to pathological conditions. Numerous studies have meticulously delineated the involvement of NRs in lipid metabolism throughout the onset and advancement of chronic diseases, thereby establishing NRs as compelling diagnostic tools and druggable targets [129–131].

## Exosomes and chronic diseases

Exosomes play a crucial role in both intra- and inter-cellular communication, first discovered in the 1980s as vesicles involved in reticulocyte maturation [35, 132–134]. The field of exosome research has experienced rapid expansion in recent years, marked by groundbreaking discoveries [135, 136]. Notably, studies revealed the predominant presence of cholesterol, sphingomyelin, ceramide, and phosphatidylserine in exosomes [137–139]. This lipid framework significantly influences various aspects of exosome dynamics such as secretion, structural integrity, cargo loading, endocytosis, and signaling processes [42, 43]. The formation of exosomes begins with their initial synthesis as intraluminal vesicles through inward budding of multivesicular bodies. Subsequently, during the maturation process from early to late endosomes, these multivesicular bodies fuse with the plasma membrane, resulting in the release of encapsulated intraluminal vesicles into the extracellular space, thereby acquiring the designation as exosomes [140]. Numerous studies have demonstrated the significant involvement of exosomes in biological processes such as cellular communication, reproduction, development, and immune response. The complex signaling pathways in cellular communication through exosomes include horizontal transfer of cargos [141]. Moreover, various signaling pathways essential for human reproduction, pregnancy and embryonic development are directly linked to exosomes [142]. Exosomes also play a vital role in sperm epididymal maturation, contributing to the production of male gametes with optimal motility [143]. Seminal exosomes from different donors exhibited   let7 family members as the most abundant miRNA that regulate interleukin (IL)-10 and IL-13 expression, suggesting a potential role of exosomes in genitalia resident immunity [144]. Further, antigen presentation during an immune response is another important function of exosomes. For example, B cell-derived exosomes carrying major histocompatibility complex class II contribute to the maintenance of T cell memory and tolerance [46]. Apart from this, they also aid in preventing placental infection through the delivery of exosomal miRNA, and breast milk-derived exosomes serve as an immune booster [145, 146].

The growing body of empirical evidence strongly supports the involvement of exosomes in the pathogenesis of various diseases as well. For example, Wen et al. [136] demonstrated that serum exosomes sourced from individuals with unstable plaque atherosclerosis (UA) exhibited an upregulation of circRNA-0006896 in human umbilical vein endothelial cells (HUVECs), compared to serum exosomes derived from patients with stable plaque atherosclerosis (SA). This upregulation of circRNA-0006896 led to subsequent binding and downregulation of miR-1264 within the HUVECs, increasing phosphorylated STAT3 and DNA methyltransferase 1 (DNMT1), ultimately inducing hypermethylation of suppressor of cytokine signaling 3 [136]. These events resulted in enhanced proliferation and migration of HUVECs, suggesting that serum exosomes from UA patients are pivotal factors in the initiation of pathological plaque formation. The underlying circRNA-0006896/STAT3/DNMT1 axis emerges as a potential novel therapeutic target for atherosclerosis [136]. In another study, Yang et al. [147] revealed that treatment with exosomes derived from mesenchymal stem cells (MSCs) containing miR-145 suppressed junction adhesion molecule A, thereby reducing the migration of HUVECs treated with oxidized low-density lipoprotein (LDL). Importantly, the study demonstrated that miR-145-enriched exosomes contributed to the reduction of plaque formation in atherosclerotic mouse models, highlighting the potential role of exosomes in preventing atherosclerosis [147]. Moreover, MSC-derived exosomes containing miR-let7 were found to promote M2 polarization and inhibit macrophage infiltration respectively through the miR-let7/high mobility group AT-hook 2/NF-κB pathway and miR-let7/insulin-like growth factor 2 mRNA-binding protein 1/phosphatase and tensin homolog (PTEN) pathways. This dual mechanism hindered the progression of atherosclerosis in both in vitro and in vivo models [135]. Additional insights into the intricate interplay between exosomes and endothelial cells were provided by Taverna et al. [148]. Exosomes derived from LAMA8A cells were observed to enhance cell-to-cell adhesion, migration, and angiogenesis in HUVECs by upregulating intercellular adhesion molecule 1 (ICAM1), vascular cell adhesion molecule 1 (VCAM1), as well as inducing phosphorylation of MAPK. These exosomes were also found to expedite the attachment of chronic myeloid leukemia cells to HUVECs, highlighting the importance of exosome-mediated communication between cancer cells and endothelial cells in promoting tumor angiogenesis [148]. Furthermore, Fuchs et al. [149] elucidated a crucial role for subcutaneous abdominal adipose tissue-derived exosomes and plasminogen activator inhibitor-1 (PAI-1) in the development of obesity. Elevated levels of both circulating exosomes and PAI-1 were detected in patients with obese NAFLD compared to obese individuals with normal intrahepatic triglyceride levels as well as a lean control group [149]. Notably, exosomes derived from the obese NAFLD group induced insulin resistance in hepatocytes and myotubes, as evidenced by reduced Akt phosphorylation [149].

Accumulating evidence indicates an upregulation of exosome production in chronic kidney diseases, leading to target various kidney cell types such as interstitial fibroblasts, tubular epithelial cells, macrophages, and endothelial cells. This subsequently modulates their behavior and function [150, 151]. For instance, Liu et al. [150] demonstrated that TGF-β-stimulated human kidney tubular cells exhibit an increase in exosome release along with a protective effect on renal interstitial cells. Exosomes containing tumor necrosis factor alpha (TNF-α) induced protein were also found to inhibit fibroblast apoptosis by degrading p53 via ubiquitination [150]. Additionally, ovariectomy results in decreased serum levels of estrogen and progesterone, accompanied by reduced urine output, increased urinary protein excretion, elevated serum creatinine and blood urea nitrogen levels, ultimately leading to renal dysfunction and fibrotic alterations that confirms chronic kidney disease. Nevertheless, therapy with exosomes derived from bone marrow mesenchymal stem cells (BMSCs) showed protective effect against these pathologies in ovariectomized rats [152]. Similarly, exosomes derived from MSCs enriched with miR-21a-5p improved unilateral ureter obstruction-induced renal fibrosis by inhibiting glycolysis in tubular epithelial cells [153]. Conversely, another study illustrated that miR-21 present in exosomes derived from TGF-β1-stimulated tubular epithelial cells accelerated fibrosis and unilateral ureter obstruction both in vitro and in vivo through PTEN/Akt signaling in obstructed kidney [51].

A recent study examined the impact of exosomes derived from BMSCs on TGF-β-induced human renal proximal tubular epithelial cells and 5/6 subtotal nephrectomy rat models [154]. These exosomes exhibited a protective effect by improving renal function, and reducing fibrotic regions, which was further enhanced when combined with si-Smurf-2 (SMAD specific E3 ubiquitin protein ligase 2) [154]. In summary, the aforementioned studies suggest the significant involvement of exosomes in modulating chronic kidney diseases, highlighting their potential to alleviate such conditions through diverse molecular targets. However, comprehensive research is necessary to validate these protective effects due to conflicting outcomes in certain studies.

The potential utility of exosomes as biomarkers in clinical settings has been demonstrated by several studies. For instance, Zhu et al. [155] reported an increase in tRNA-derived small RNAs in plasma exosomes isolated from liver cancer patients compared to healthy donors, suggesting their potential as diagnostic biomarkers for liver cancer. Additionally, Jiao et al. [156] showed that exosomes derived from chronic hepatitis B and acute-on-chronic liver failure patients exhibited enhanced expression of CD63 and albumin compared to survival group, with a higher percentage of these exosomes in acute-on-chronic liver failure compared to chronic hepatitis B. Notably, the study indicated that albumin and vascular endothelial growth factor (VEGF) present in exosomes could serve as biomarkers for liver regeneration and prognostic evaluation [156]. These collective findings contribute to the understanding of the diverse roles of exosomes in various disease pathologies, emphasizing their potential as both diagnostic biomarkers and therapeutic targets.

In conclusion, these investigations collectively shed light on the intricate functions of exosomes in modulating diverse cellular signaling pathways, including their regulatory influence on STAT3, the epigenetic regulatory mechanisms mediated by DNMT1, and the complex signaling cascades orchestrated by non-coding RNA. These delineated mechanisms play crucial roles in the development of chronic diseases. Furthermore, the identification of exosomes expressing modified surface markers emerges as a significant approach, proving beneficial not only for disease diagnosis but also for identifying novel therapeutic targets for treating chronic diseases.

## Role of exosomes in modulating NRs

**Fig. 3:**
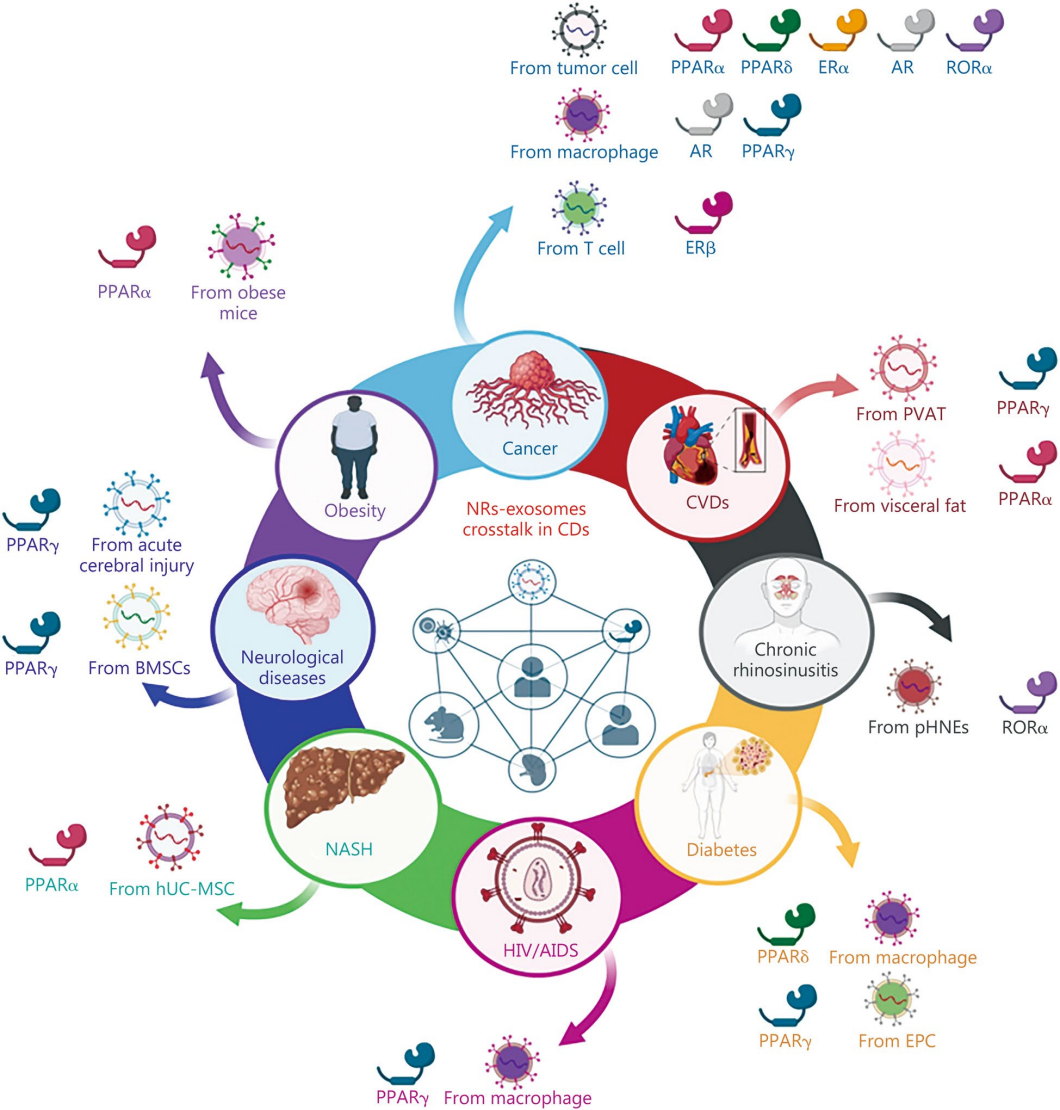
Intercellular communication mediated by nuclear receptors (NRs) and exosomes in the pathophysiology of chronic diseases. The intricate network of intercellular communication facilitated by NRs and exosomes, elucidating their pivotal role in the etiology and progression of chronic diseases such as cancer, cardiovascular diseases (CVDs), chronic rhinosinusitis, diabetes, HIV/AIDS, non-alcoholic steatohepatitis (NASH), neurological diseases, and obesity. In the context of these chronic maladies, the interplay between NRs and exosomes emerges as a critical determinant, influencing disease development and trajectory. This figure illustrates the sources of exosomes and their reported target NRs across the spectrum of chronic diseases under consideration. A comprehensive understanding of these underlying mechanisms holds promise for identifying novel therapeutic targets, thereby paving the way for innovative treatment modalities for a myriad of chronic diseases. AIDS acquired immune deficiency syndrome, AR androgen receptor, BMSC bone marrow mesenchymal stem cell, EPC endothelial progenitor cell, ER estrogen receptor, HIV human immunodeficiency virus, hUC-MSC human umbilical cord mesenchymal cell, MDSC myeloid derived suppressor cell, PPAR peroxisome proliferator-activated receptor, PVAT perivascular adipose tissue, RORα retinoic acid receptor related orphan receptors alpha

**Fig. 4:**
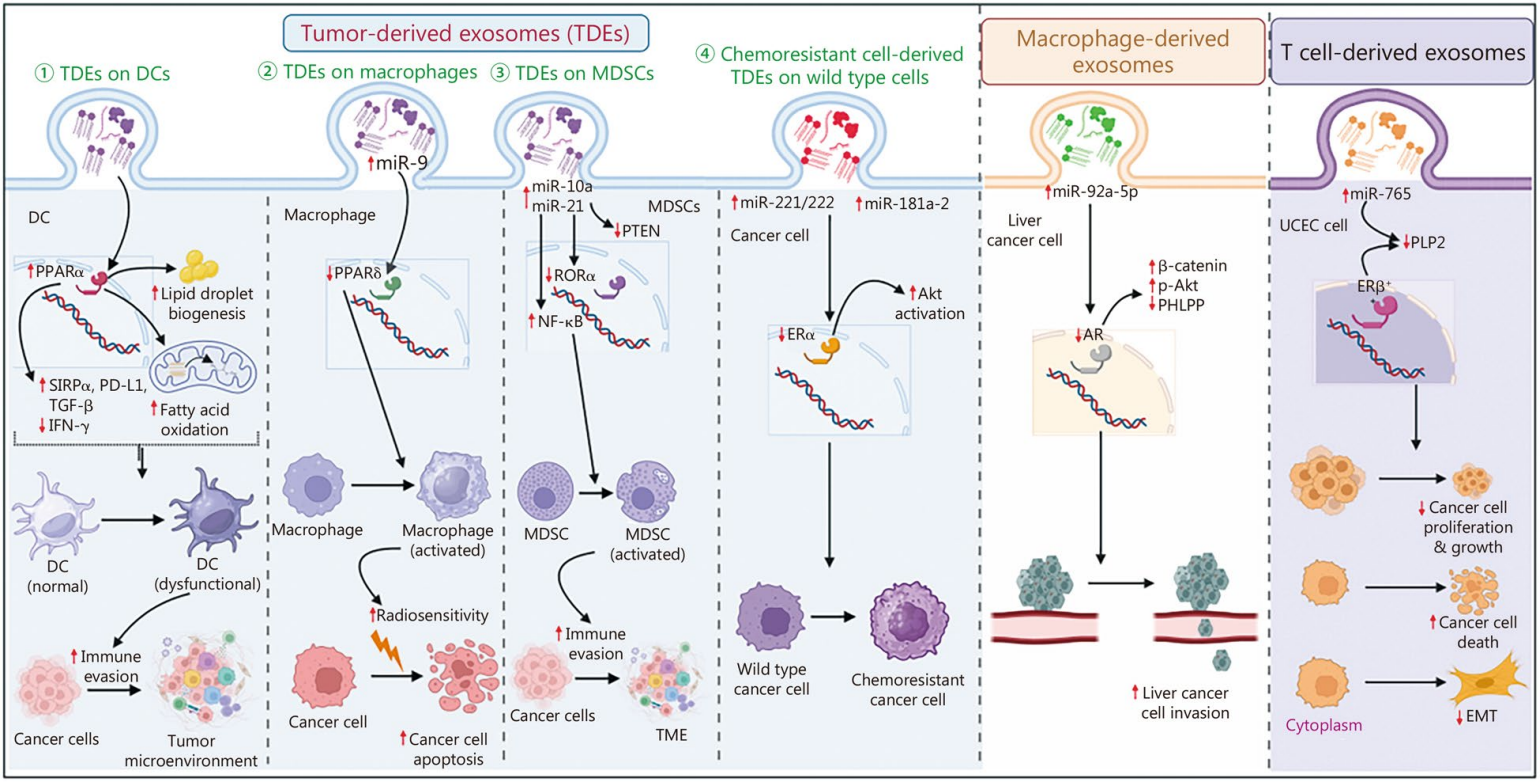
Interplay between exosomes and nuclear receptors (NRs) in regulating tumor microenvironment dynamics. This figure illustrates the intricate cross-talk between exosomes and NRs in cancer pathogenesis. Tumor-derived exosomes (TDEs) convey genetic material, including genes and non-coding RNAs, to dendritic cells (DCs) and myeloid-derived suppressor cells (MDSCs). This molecular cargo targets specific NRs, influencing the functionality of these immune cells and contributing to immune evasion. TDEs from HPV^+^ HNSCC cells downregulate PPARδ, activating macrophages and enhancing radiosensitivity. Chemoresistant cancer cells use exosomes to transfer miRNAs targeting NRs to wild-type cancer cells, conferring therapeutic resistance. Macrophage-derived exosomes regulate the AR/PHLPP/Akt/β-catenin axis in liver cancer cells, promoting invasion. Conversely, T cell-derived exosomes deliver miR-765, suppressing PLP2 in ERβ^+^ uterine corpus endometrial carcinoma (UCEC) cells, reducing proliferation, EMT, and inducing apoptosis. These findings highlight the communication network between cancer cells and stromal cells within the tumor microenvironment via exosomal/NR interactions, suggesting that manipulating NR expression through targeted interventions presents a promising therapeutic strategy for diverse cancer types. Akt protein kinase B, CDs chronic diseases, EMT epithelial to mesenchymal transition, ERβ estrogen receptor β, IFN-γ interferon-γ, miR microRNA, NF-κB nuclear factor kappa-B, PD-L1 programmed death-ligand 1, PHLPP PH domain and leucine rich repeat protein phosphatases, PLP2 proteolipid protein 2, PPAR peroxisome proliferator-activated receptor, PTEN phosphatase and tensin homolog*,* RORα retinoic acid receptor related orphan receptor alpha, SIRPα signal regulatory protein alpha, TGF-β transforming growth factor-β, TME tumor microenvironment

In recent years, the phenomenon of intercellular communication across distant cellular entities through paracrine signaling, particularly via exosome-mediated pathways, has attracted increased attention due to its crucial involvement in both physiological and pathological contexts [136]. Exosomes, in particular, play a regulatory role in manipulating various proteins within recipient cells, including NRs [29, 157]. The following discussion will outline the mechanism of the interaction between exosomes and NRs, with implications for pathophysiological functions. The involvement of exosomes and their contents that target NRs in the pathogenesis of chronic diseases has been summarized in Table 1 [29–31, 157–177], Figs. 3 and 4 [162, 163, 165, 166, 170].

**Table 1:** Molecular mechanism underlying exosomes targeting nuclear receptors (NRs) in the pathogenesis of chronic diseases

Disease	NR	Exosomal content	In vitro/in vivo/clinical	Cell line/model	Intervention/treatment/expression modulation	Mechanism of action/outcome	References
ACI	PPARγ	miR-27-3p	In vitro	Microglia stimulated with LPS	miR-27-3p mimic	↓PPARγ ↑IL-1β, IL-6, TNF-α, Iba1	[158]
					miR-27-3p inhibitors	↑PPARγ ↓IL-1β, IL-6, TNF-α, Iba1	
			In vivo	MCAO rats	Serum exosomes from ACI patients	↓PPARγ, neurological score ↑Cerebral injury, IL-1β, IL-6, TNF-α, microglial cells, macrophages, foot fault	
					Serum exosomes from ACI patients + miR-27-3p inhibitor	↓IL-1β, IL-6, TNF-α, Iba1	
					PPARγ over expression + serum exosomes from ACI patients	↓IL-1β, IL-6, TNF-α, Iba1	
Atherosclerosis	PPARγ	miR-382-5p	In vitro	RAW264.7	miR-382-5p inhibitor-PVAT-EXO	↑Foam cell formation, BMP4 ↓Dil-oxLDL uptake, cholesterol efflux, ABCA1, ABCG1, PPARγ, SR-A	[159]
					BMP4 + PVAT-EXO	↑Foam cell formation ↓Cholesterol efflux, ABCA1, ABCG1, PPARγ, SR-A	
					siPPARγ + Noggin	↑Foam cell formation ↓Cholesterol efflux, ABCA1, ABCG1	
	PPARα	miR-27b-3p	In vitro	HUVECs	miR-27b-3p mimic + TNF-α	↑p-p65, VCAM1, ICAM1, MCP1	[160]
					CM from 3T3-L1 transfected with miR-27b-3p mimic	↑p-p65, IL-1β, IL-6, VCAM1, ICAM1, MCP1	
					CM from 3T3-L1 transfected with miR-27b-3p inhibitor	↓p-p65, IL-1β, IL-6, VCAM1, ICAM1, MCP1 ↑PPARα	
					CM from 3T3-L1 transfected with miR-27b-3p mimic + GW4869	↓IL-1β, IL-6, VCAM1, ICAM1, MCP1	
					PPARα (OE) + miR-27b-3p mimic	↓IL-1β, IL-6, VCAM1, ICAM1, MCP1	
			In vivo	ApoE^−/−^ mice	miR-27b-3p mimic	↑Weight, atherosclerotic lesions, collagen, lipid accumulation in aortic sinus, VCAM1	
					PPARα (OE)	↓VCAM1, atherosclerotic lesions, aortic sinus plaques	
Burn injury	PPARγ	LncRNA H19	In vitro	PBMC differentiated macrophages	EXO-shH19	↑TNF-α, IL-1β, proliferation, miR-130b-3p ↓IL-10, VEGF-A, arginase, CD206	[31]
					EXO-shH19 + anti-miR-130b-3p	↓TNF-α, IL-1β ↑IL-10, VEGF-A, arginase, CD206	
					Anti-miR-130b-3p	↑PPARγ, STAT3	
				HSF	EXO-shH19	↓Migration, proliferation	
					EXO-shH19 + anti-miR-130b-3p	↑Migration, proliferation	
				HUVECs	EXO-shH19	↓Tube formation	
					EXO-shH19 + anti-miR-130b-3p	↑Tube formation	
			In vivo	BALB/c mice	Exo-shH19	↑Fibrosis, TNF-α, IL-1β ↓Wound closure CD31^+^ area, CD163^+^ area, PPARγ, STAT3, VEGF-A, IL-10	
					EXO-shH19 + anti-miR-130b-3p	↑Wound closure, CD31^+^ area, CD163^+^ area, PPARγ, STAT3, VEGF-A, IL-10 ↓Fibrosis, TNF-α, IL-1β	
Cancer							
Breast cancer	ERα	miR-221/222	In vitro	MCF-7	Exosomes from Tam resistant MCF-7 cells	↓ERα, p27	[161]
					Exosomes from Tam resistant MCF-7 cells + anti-miR-221/222	↓Cell viability, colony formation ↑Apoptosis, ERα, p27	
	ERα	miR-181a-2	In vitro	MCF-7	Tamoxifen	↑miR-181a-2	[162]
					miR-181a-2 mimics	↓ERα ↑p-Akt, chemoresistance, cell viability	
					MCF-7T exo	↓ERα ↑p-Akt	
Cervical cancer	PPARα	FAs	In vitro	BMDCs	Tumor derived exosomes	↑Body lipid, SIRPα, PD-L1, TGF-β, Treg, mitochondrial mass, OCR, FAO, PPARα*,* ATP production ↓Antigen presentation, CD8^+^ T cells, IFN-γ, lactate, ECAR	[163]
					Tumor derived exosomes + GW6471	↑ECAR, lactate, IFN-γ ↓OCR, respiration, ATP production, FAO, Treg	
			In vivo	TC-1 xenografts	GW6471 + E7 vaccine	↑Survival ↓Tumor volume	[163]
Colon cancer	PPARα	FAs	In vivo	MC38-OT I xenografts	GW6471	↓Lipid in TIDC	[163]
				MC38-OT I xenografts	GW6471 + PD-L1 mAb	↑CD8^+^ T cells, IFN-γ, survival ↓Tumor volume	
				PPARα^−/−^ MC38-OT I xenografts	–	↓Lipid in TIDC, tumor volume ↑CD8^+^ T cells, IFN-γ	
Glioma	RORα	miR-10a & miR-21	In vitro	P3GDEs/GL261GDEs	MDSCs	↑Uptake of exosomes	[164]
				BM cells	h-GDEs from the culture supernatants of U87, P3, GL261, and G422	↑Gr-1^+^CD11b^+^ MDSCs, *IL-10* & *TGF-β* in MDSCs	
					GM-CSF/IL-6 + GDEs (N + 10/N + 21) GL261/G422	↑Gr-1^+^CD11b^+^	
					GM-CSF/IL-6 + GDEs (H10KO/H21KO) GL261/G422	↓Gr-1^+^CD11b^+^	
					GM-CSF/IL-6 + siRORα/siPTEN	↑Gr-1^+^CD11b^+^	
					GM-CSF + TNF-α + N10GDEs from GL261/G422	↓RORα, IκBα ↑p65 in nucleus	
				MDSCs from BM cells	GM-CSF/IL-6 + siPTEN	↑p-STAT3, p-Akt ↓PTEN, p-p65	
				MDSCs from glioma mice	h-GDEs from GL261, and G422 cells	↓*RORα, PTEN*	
			In vivo	C57BL/6 mice	GDEs	↑Uptake of exosomes	[164]
					h-GDEs from GL261 and G422 cells	↑Gr-1^+^CD11b^+^	
					Gr-1^+^CD11b^+^ MDSCs with splenic T cells	↓T cell proliferation ↑ROS, NO, arginase, *IL-10*, *TGF-β*	
				Glioma xenografts (GL261 cells)	miR-10a-5p, miR-21-5p	↑ROS, *IL-10*, *TGF-β* ↓*IL-12*	
				C57BL/6 mice	N + 21 GDEs/N + 10 GDEs of GL261/G422	↑Gr-1^+^CD11b^+^	
					Gr-1^+^CD11b^+^ MDSCs with splenic T cells	↑T cell proliferation ↓ROS, NO, arginase, *IL-10*, *TGF-β*	
				C57BL/6 mice	H + 10KO GDEs/H + 21KO GDEs of GL261/G422	↓Gr-1^+^CD11b^+^	
					Gr-1^+^CD11b^+^ MDSCs with splenic T cells	↓T cell proliferation ↑ROS, NO, arginase, IL-10, TGF-β	
				Glioma xenografts (GL261 cells)	miR10KO	↓Tumor volume, miR10a, Gr-1^+^CD11b^+^	
					Gr-1^+^CD11b^+^ MDSCs with splenic T cells	↑T cell proliferation ↓ROS, NO, arginase, IL-10	
				Glioma xenografts (GL261 cells)	miR10KO	↓Tumor volume, miR21, Gr-1^+^CD11b^+^	
					Gr-1^+^CD11b^+^ MDSCs with splenic T cells	↓Arginase	
				Glioma xenografts (GL261 cells)	h-GDEs from GL261, and G422 cells	↓RORα, PTEN in MDSCs	
					MDSCs with siRORα	↑ROS, IL-10, TGF-β	
					MDSCs with siPTEN	↑IL-10	
				MDSCs from Glioma mice	siPTEN	↑p-STAT3, p-Akt ↓PTEN, Vpp65	
HCC	AR	miR-92a-5p	In vitro	HA22T/SK-HEP-1	Thp1 derived exosomes	↓AR, PHLPP ↑Invasion	[165]
				HA22T	Thp1 derived exosomes	↑β-catenin, p-Akt ↓PHLPP1	
				HA22T	Thp1 derived exosomes + over expression of miR-92a-5p	↑β-catenin, p-Akt, invasion ↓AR	
				SK-HEP-1	Thp1 derived exosomes + anti-miR-92a-5p	↓β catenin, p-Akt	
				HA22T/SK-HEP-1	Thp1 derived exosomes + GW4869	↑AR ↓Invasion	
				HA22T/SK-HEP-1	Over expression of miR-92a-5p	↓AR ↑Invasion	
			In vivo	Male nude mice xenograft (SK-HEP-1)	Thp-1-PLKO cells	↓AR ↑Tumor, metastasis	
HNSCC	PPARδ	miR-9	In vitro	SCC47, SCC104, SCC90	HPV infection	↑Exosomes secretion	[166]
				SCC47, SCC104, SCC90	THP1 exosomes derived from HNSCC treated with HPV	↑iNOS, IL-6, TNF-α ↓CD163, IL-10, radio sensitivity	
				SAS, CAL33	THP1 exosomes derived from HNSCC without HPV treatment	↓iNOS, IL-6, TNF-α ↑CD163, IL-10	
				THP1	miR-9 mimic	↓PPARδ	
MB	PPARγ	↓Let-7i-5p, miR-221-3p	In vitro	RAW264.7+ Daoy cells	-	↓IL-1β, TNF-α ↑Arg1, Mrc1, IL-10, TGF-1β	[167]
				RAW 264.7	Daoy exosomes	↓IL-1β ↑Arg1, iNOS, IL-10	
					Daoy exosomes + Let-7i-5p mimic, miR-221-3p mimic	↓PPARγ	
					AntimiRs + siPPARγ	↓TNF-α ↑IL-10, TGF-1β	
			In vivo	NeuroD2:SmoA1 mice	GW9662 + Sonidegib	↓Tumor volume, PPARγ, TAMs ↑Survival, microglial cells	
Osteosarcoma	RORα	miR-181a-5p	In vitro	THP-1	Exosomes from SAOS2 cells	↑CD11b, CD163, CD206, IL-10	[168]
					Exo-anti-miR-181a-5p	↓CD11b, CD163, CD206, IL-10 ↑RORα	
					Exo-anti-miR-181a-5p + siRORα	↑CD11b, CD163, CD206, IL-10 ↓RORα	
			Clinical	Osteosarcoma patients’ tissue	–	↓RORα	
Prostate cancer	AR	–	In vitro	LNCaP cocultured with PC-3 in androgen deprived medium	–	↓AR, PSA, Cdk1, Cdk2 ↑Cell viability, S phase cells	[169]
				LNCaP	PC-3 derived exosomes	↓AR, PSA, G1 phase ↑Cell viability, S phase cells, HMOX1	
					Hemin	↑HMOX1, cell viability	
			In vivo	Castrated NOD/SCID mice	LNCaP cultured with PC-3 exosomes	↑Tumorigenicity	
			clinical	AIPC tissue sample	–	↑HMOX1	
UCEC	ERβ	miR-765	In vitro	KLE/Ishikawa/RL95-2	–	↓miR-765	[170]
				Ishikawa	miR-765 mimic	↑Cell viability, Ki67, TJP1 ↓COL3A1, FN1, CDH1, CDH2, S100A, MMP9, SNAIL, ZEB1, PLP2	
				KLE/Ishikawa	siPLP2	↓Viability, invasion, vimentin, COL3A1, FN1, S100A, SNAIL, ZEB1 ↑TJP1, E-cadherin	
					Estrogen	↓miR-765 ↑PLP2	
					Estrogen + PHTPP/Fulvestrant	↑miR-765 ↓PLP2	
					Estrogen + miR-765 mimic	↓PLP2	
				Ishikawa	CD45RO-CD8^+^ T cell-derived exosomes	↑miR-765 ↓PLP2	
					Over expression of PLP2	↑Notch1, NID, Hes1, vimentin, invasion, viability ↓E-cadherin	
					CD45RO-CD8^+^ T cell-derived exosomes + estrogen	↑miR-765, E-cadherin, ↓PLP2, viability, Ki67, vimentin, invasion,	
			In vivo	Ishikawa xenograft	miR-765 mimic	↓Tumor volume ↑Survival	
					siPLP2	↓Metastasis ↑Survival	
					CD45RO-CD8^+^ T cell-derived exosomes + estrogen	↓Tumor volume ↑Survival	
			Clinical	Patients tissue	–	↓miR-765	
CRS	RORα	miR-19a, miR-614	In vitro	RPMI 2650	Air particulate matter	↓Cell viability, DC SIGN ↑TNF-α, IL-1β, IL-6, miR-19a, miR-614	[29]
				pHNE exosome	Air particulate matter	↑miR-19a, miR-614	
				M0 macrophage	miR-19a, miR-614	↓DC SIGN ↑TNF-α, IL-1β, IL-6,	
					Exosomes from pHNE	↓RORα	
					miR-19a/miR-614 inhibitors	↑RORα	
					siRORα	↑CCL2, CCL5, MIP-1, CXCL1, CXCL11, TNF-α, IL-1β, MIF, PAI-1	
			Clinical	CRC patient tissue	–	↓RORα ↑TNF-α, IL-1β, IL-6, miR-19a, miR-614	
Diabetic wound	PPARγ	miR-182-5P	In vitro	HaCaT	High glucose	↓Cell viability, cell adhesion, collagen 4, hyaluronan, clone formation, FN1, CTNNB1 ↑Cell cycle arrest, PPARγ, MMP1	[171]
					High glucose + EPC-exo	↑Cell viability, cell adhesion, collagen-4, hyaluronan, clone formation ↓Cell cycle arrest	
					High glucose + has-miR-182-5P	↓PPARγ, MMP1 ↑FN1, CTNNB1	
					siPPARγ	↑Cell viability, cell adhesion, Collagen-4, hyaluronan, clone formation, FN1 ↓PPARγ, MMP1	
					siPPARγ + miR-182-5P-inhibitor	↓Cell viability, cell adhesion, collagen- 4, hyaluronan, clone formation, FN1 ↑PPARγ, MMP1	
			In vivo	C57BL/6J diabetic mice	EPC-exo	↑Wound healing	
				C57BL/6J diabetic mice	has-miR-182-5P-overexpression	↑Wound healing	
HIV	PPARγ	miR-27a, miR-23a, miR-115, miR-21	In vitro	THP1, MDM	HIV proteins	↑miR-27a, miR-23a, miR-115, miR-21	[172]
				BEAS-2B	Antagomir-exo	↓ZO-1	
					Tat treated macrophage-exos	↓PPARγ, basal respiration, spare respiratory capacity, maximal respiration, and ATP turn over ↑GLUT1, glucose uptake, glycolytic capacity	
			In vivo	HIV transgenic rats	–	↑miR-27a, miR-23a, miR-115, miR-21	
Liver I/R injury	PPARδ	miR-122-5P	In vitro	RAW264.7	EVs	↑iNOS, TNF-α, IL-6, Arg1	[173]
					Ago-miR	↑iNOS, TNF-α, IL-6, Arg1, p-p65 ↓PPARδ	
					siPPARδ	↑iNOS, TNF-α, IL-6, Arg1, p-p65 ↓PPARδ	
			In vivo	C57BL/6 mice	EVs	↑Liver injury, suzukis score, iNOS, TNF-α, IL-6, ALT, F4/80^+^CD11b^−^CD86^+^, F4/80^+^iNOS^+^	
				IR6h mice	GW4869	↓Suzukis score, iNOS, IL-6, ALT, F4/80^+^iNOS^+^	
				IR6h mice	AgomiR	↑Liver injury, suzukis score, *iNOS, TNF-α, IL-6*, ALT, F4/80^+^CD11b^−^CD86^+^, F4/80^+^iNOS^+^	
				IR6h mice	AntagomiR	↓Liver injury, suzukis score, *iNOS, TNF-α, IL-6*, ALT, F4/80^+^iNOS^+^	
			Clinical	Patient after liver transplantation	–	↑miR-122-5P	
NASH	PPARα	–	In vitro	HEPG2	PA + Exo	↑ROS, p-Nrf2, NQO1	[30]
					PA + Exo + ML385	↓p-Nrf2, NQO1	
				AML2	MCD + Exo	↑p-Nrf2, NQO1, ROS	
					MCD + Exo + ML385	↓p-Nrf2, NQO1	
			In vivo	C57BL/6 J HFHC/MCD mice	hUC-MSCs exosomes	↓NAS score, ALT, TG, TNF-α, IL-6, F4/80, CD11C, SREBP1C, FAS, MDA, CYP2E1 ↑CD206, PPARα, FABP5, ACOX, CPT1α, p-Nrf2, NQO1, p-AMPK, SOD, GSH	
	PPARα	–	In vitro	RAW264.7	Ox-LDL + UC-MSC exosome	↑IL-10, arginase, CD 206 ↓TNF-α, IL-1β, IL-6, lipid droplet/cell number	[174]
				Huh1-6/HepRG	Ox-LDL + CM	↓PPARα	
					Ox-LDL + UC-MSC exosome	↑PPARα	[174]
			In vivo	MCD mice	–	↓Body weight, liver weight, PPARα ↑NAS score, ALT, AST, TNF-α, IL-1β, IL-6, steatosis, p-p65	
					UC-MSC exosome	↑Body weight, liver weight, IL-10, arginase, CD206, PPARα ↓NAS score, ALT, AST, TNF-α, IL-1β, IL-6, steatosis, p-p65	
Obesity	PPARδ	miR-29a	In vitro	3T3-L1 adipocytes/L6 myocytes/primary hepatocytes	Obese-ATM-exo	↑miR-29a	[157]
				3T3-L1 adipocytes/L6 myocytes	Insulin + miR-29a mimic	↓Glucose uptake, PPARδ	
				Primary hepatocytes	Insulin + miR-29a mimic	↓PPARδ ↑Glucose output	
				3T3-L1 adipocytes/L6 myocytes	GW501516 + insulin + miR-29a mimic	↑Glucose uptake	
				Primary hepatocytes	GW501516 + insulin + miR-29a mimic	↓Glucose output	
			In vivo	C57BL/6 J lean mice	Antagomir-29a-obese-ATM-exo	↓Fasting blood glucose, fasting serum insulin, insulin resistance	
	PPAR	miR-192, miR-122, miR-27α-3p, miR-27b-3p	In vitro	3T3-L1	–	↓PPAR	[175]
			In vivo	C57/6J mice with HFD	Exosomes	↑Glycemia, insulin tolerance, PPARγ in liver, PPARα in liver, *Ccl2* in eWAT ↓PPARγ in eWAT, PPARα in eWAT	
					Mimic + FF	↓TG, glucose intolerance, FFA	
				C57/6J mice	mimic-miRNA-exo	↑TG, FFA, glycemia, insulin tolerance, PPARγ in liver, PPARα in liver, *Ccl2* in eWAT, macrophage infiltration, lipid droplet ↓PPARγ in eWAT, PPARα in eWAT	
					siPPARα-exosome	↓PPARα in eWAT, mitochondrial content in eWAT ↑*Ccl2* in eWAT, glycemia, FFA in liver, PPARγ in liver	
Parkinson’s disease	PPARγ	Wnt	In vivo	Wistar rats	6OHDA	↓Sleep time, slow wave sleep time, fast wave sleep time, dopamine, 5-HT, PPARγ, Clock, Bmal1, Per2, Wnt-5a ↑Awaken time, mitochondrial membrane potential	[176]
					6OHDA + BMSC^induced^ exo	↑Sleep time, slow wave sleep time, fast wave sleep time, dopamine, 5-HT, PPARγ, Clock, Bmal1, Per2, Wnt-5a ↓Awaken time, mitochondrial membrane potential	
PH	PPARγ	miR-211	In vitro	PASMC	miR inhibitor	↑CAMK1, PPARγ ↓Proliferation	[177]
					Overexpression of CAMK1	↓PPARγ	
			In vivo	SD rats	Hypoxia	↓CAMK1, PPARγ ↑miR-211	
					miR-211 overexpression exo	↑miR-211, mPAP, wall area, wall thickness ↓CAMK1, PPARγ,	
					Hypoxia + miR-211 inhibitor	↑CAMK1, PPARγ ↓miR-211, mPAP, wall area, wall thickness	

*5-HT* 5-hydroxytryptamine, *6OHDA* 6-hydroxydopamine, *ABCA1* ATP-binding cassette A1, *ABCG1* ATP binding cassette G1, *ACI* acute cerebral injury, *ACOX1* acyl-CoA oxidase 1, *ALT* alanine transaminase, *AMPK* AMP-activated protein kinase, *ApoE* apolipoprotein E, *AR* androgen receptor, *AST* aspartate aminotransferase, *ATP* adenosine triphosphate, *Arg1* arginase 1, *Bmal1* brain and muscle ARNT-like protein 1, *BM* Bone marrow, *BMDCs* bone marrow-derived dendritic cells, *BMP4* bone morphogenetic protein, *CAMK1* calcium/calmodulin dependent protein kinase I, *CCL2* chemokine (C–C motif) ligand 2, *CD206* cluster of differentiation 206, *Cdk* cyclin-dependent kinase, *Clock* Circadian locomotor output cycles protein kaput, *CDH1* cadherin 1, *CDH2* cadherin 2, *CM* conditioned media, *COL3A1* collagen type III alpha 1 chain, *CPT1α* carnitine palmitoyl transferase I, *CRS* chronic rhinosinusitis, *CTNNB1* catenin beta 1, *CXCL1* C-X-C motif chemokine ligand 1, *CXCL11* C-X-C motif chemokine ligand 11, *CYP2E1* cytochrome P450 2E1, *DC* dendritic cell, *Dil-oxLDL* oxidized low-density lipoprotein, *ECAR* extracellular acidification rate, *ER* estrogen receptor, *EV* extracellular vesicle, *eWAT* epididymal white adipose tissue, *FF* free fatty acids, *FAs* fatty acids, *FABP5* fatty acid-binding protein 5, *FAO* fatty acid oxidation, *FAS* fatty acid synthase, *FFA* free fatty acid, *FN1* fibronectin, *GDEs* glioma-derived exosomes, *GLUT1* glucose transporter 1, *GM-CSF* granulocyte–macrophage colony-stimulating factor, *GSH* glutathione, *HCC* hepatocellular carcinoma, *h-GDEs* hypoxic glioma-derived exosomes, *Hes1* hes family bHLH transcription factor 1, *HIV* human immunodeficiency virus, *HNSCC* head and neck squamous cell carcinoma, *HMOX1* heme oxygenase 1, *HPV* human papilloma virus, *HSF* human skin fibroblast, *HUVECs* human umbilical vein endothelial cells, *Iba1* ionized calcium-binding adaptor molecule 1, *ICAM1* intercellular adhesion molecule 1, *IFN-γ* interferon-γ,* I*L interleukin, *iNOS* inducible nitric oxide synthase, *IκBα* inhibitor of nuclear factor kappa B, *lncRNA* long non-coding RNA, *LPS* lipopolysaccharide, *LRP* low-density lipoprotein (LDL) receptor-related protein, *LXR* liver X receptor, *mAb* monoclonal antibody, *MB* medulloblastoma, *MCAO* middle cerebral artery occlusion, *MCD* methionine-choline deficient, *MCP1* monocyte chemoattractant protein 1, *MDA* malondialdehyde, *MDM* human monocyte-derived macrophages, *MDSC* myeloid-derived suppressor cell, *MIP-1* macrophage inflammatory protein-1, *miR* microRNA, *MMP* matrix metalloproteinase, *Mrc1* mannose receptor C type 1, *mPAP* mean pulmonary arterial pressure, *MRP* multidrug resistance associated protein, *MIF* macrophage migration inhibitory factor, *NAS* NAFLD activity score, *NASH* nonalcoholic steatohepatitis, *NF-κB* nuclear factor kappa-B, *NID* nidogen 1, *NQO1* NAD(P)H quinone dehydrogenase 1, *Nrf2* nuclear factor erythroid 2-related factor 2, *OC* osteoclast, *OCR* oxygen consumption rate, *OE* over expression, *PA* palmitic acid, *PAI-1* plasminogen activator inhibitor-1, *PASMC* pulmonary artery smooth muscle cell, *PBMC* peripheral blood mononuclear cell, *PD-L1* programmed death-ligand 1, *Per2* period circadian regulator 2, *P-gp* p-glycoprotein, *PH* pulmonary hyper tension, *PHLPP* PH domain and leucine rich repeat protein phosphatases, *PLP2* proteolipid protein 2, *PPAR* peroxisome proliferator-activated receptor, *PSA* prostate-specific antigen*, PTEN* phosphatase and tensin homolog, *PVAT-EXO* perivascular adipose tissue derived exosome, *ROR* retinoic acid receptor related orphan receptor, *ROS* reactive oxygen species, *SD* Sprague Dawley, *SIRPα* signal regulatory protein alpha, *SOD* superoxide dismutase, *SR-A* scavenger receptor-A, *SREBP-1C* sterol regulatory-element-binding protein-1c, *STAT3* signal transducer and activator of transcription 3, *TAM* tumor associated macrophage, *TG* triglycerides, *TGF-β* transforming growth factor*-β*, *Thp1* human leukemia monocytic cell line, *TIDC* tumor-infiltrating dendritic cells, *TNF-α* tumor necrosis factor-α*, Treg* regulatory T cells, *UCEC* uterine corpus endometrial carcinoma, *VCAM1* vascular cell adhesion molecule 1, *VEGF-A* vascular endothelial growth factor-A, *ZEB1* zinc finger E-box binding homeobox 1, *ZO-1* zonula occludens-1

### Cancer

Cancer stands as a predominant global public health concern, despite notable advancements in current therapeutic modalities, the treatment of cancer remains a formidable challenge due to suboptimal therapeutic efficacy attributed to insufficient specificity and diminished bioavailability [178]. Pharmaceutical agents targeting NRs have emerged as one of the most clinically efficacious inhibitors of cancer [126, 128]. Additionally, numerous investigations have provided insights into the intricacies of cancer cell signaling facilitated by exosomes, actively participating in processes such as angiogenesis, chemoresistance, extracellular matrix remodeling, immune evasion, metastasis, and related phenomena [179, 180]. This section focuses on elucidating the interplay between NRs and exosomes in various aspects of cancer biology. For instance, a study demonstrated that tumor cell-derived exosomes enriched with fatty acids induced immune dysfunction in dendritic cells (DCs), thereby promoting immune evasion [163]. These tumor-derived exosomes (TDEs) were found to induce PPARα in DCs, leading to fatty acid accumulation and oxidation, resulting in a shift towards mitochondrial oxidative phosphorylation [163]. Also, these TDEs upregulated signal regulatory protein alpha (SIRPα), programmed cell death-ligand 1 (PD-L1), and TGF-β, while downregulated interferon gamma (IFN-γ). Pharmacological inhibition or genetic deficiency of PPARα abrogated these effects and augmented the efficacy of immunotherapy [163]. Furthermore, exosomes originating from human papillomavirus (HPV)-infected head and neck squamous cell carcinoma (HNSCC) revealed elevated concentrations of miR-9. These exosomes were transported to macrophages, inducing M1 polarization through the downregulation of PPARδ and enhancing radiosensitivity in HPV^+^ HNSCC cells [166]. Moreover, The Cancer Genome Atlas data revealed upregulation in the expression of miR-9 with concurrent downregulation of PPARδ in patients with HNSCC who exhibited a complete response [166]. Therefore, targeting miR-9/PPARδ axis plays a critical role in radiosensitizing HPV^+^ HNSCC cells and may offer a novel treatment strategy for this malignancy [166]. Male prevalence of hepatocellular carcinoma (HCC) may be associated with sex hormones such as androgen and estrogen during its initiation, progression and metastasis [165]. Another study has demonstrated that exosomes derived from macrophages enhanced the invasion of liver cancer cells. Exosomal miR-92a-5p can bind to the 3’UTR of AR, leading to transcriptional repression and modulation of the AR/PH domain and leucine rich repeat protein phosphatases (PHLPP)/p-Akt/β-catenin pathway in preclinical settings [165]. Besides, treatment with antagomir reversed this effect and reduced invasion of HCC cells [165]. Guo et al. [164] showed that exosomes derived from hypoxic glioma-derived exosomes (h-GDEs) containing miR-10a and miR-21 highly induce the activation of myeloid-derived suppressor cells (MDSCs) compared to exosomes derived from normoxic glioma cells. These h-GDEs were found to target RORα and PTEN by modulating the RORα or inhibitor of nuclear factor kappa B (IκBα)/NF-κB pathway and PTEN/PI3K/Akt pathway respectively [164]. Besides, inhibition of RORα significantly upregulated p65 in nucleus while reduced IκBα, explaining intercellular communication between tumor environment and immune system through exosomes by involving hypoxia [164]. Moreover, another study revealed that miR-765 in CD45RO^−^CD8^+^ T cell-derived exosomes downregulated proteolipid protein 2 (PLP2) in uterine corpus endometrial cancer (UCEC) cells, resulting in reduced proliferation, survival, and epithelial-mesenchymal transition (EMT) [170]. Estrogen was identified as a regulator of miR-765/PLP2 through the ERβ receptor in UCEC cells [170]. Treatment with miR-765 enriched exosomes from CD45RO^−^CD8^+^ T cells alleviated estrogen-dependent tumor growth in UCEC cells bearing xenograft mouse models [170]. This indicates a mechanism of intercellular communication via exosomal RNA, whereby tumor cells affect the transcriptomes of immune cells. It provides new insights for exploring the connections between hypoxia and the cancer immune microenvironment.

The reciprocal communication between donor and recipient cells, facilitated by the transport of exosomes has been implicated in conferring chemoresistance. For example, Wei et al. [161] demonstrated that exosomes derived from tamoxifen-resistant MCF-7 (MCF-7 TamR) cells exhibit distinct RNA and protein compositions compared to exosomes from tamoxifen-sensitive wild-type MCF-7 (MCF-7wt) cells. Upon entry into MCF-7 sensitive cells, exosomes from MCF-7 TamR cells release miR-221/222, resulting in reduced expression of ERα and p27 in wild-type cells, ultimately contributing to tamoxifen resistance [161]. Another study revealed that exosomes derived from adriamycin-resistant MCF-7 (MCF-7/ADR) cells promote a drug-resistant phenotype by transferring multidrug resistance protein 1 [181]. Treatment with psoralen was shown to diminish the formation and secretion of these exosomes, thereby overcoming drug resistance in MCF-7 cells. Furthermore, the Kyoto Encyclopedia of Genes and Genomes pathway analysis implicated that PPAR and p53 pathways may be involved in exosomal formation, cargo packing, and secretion [181]. In another investigation, miRNA profiling of exosomes derived from MCF-7wt cells and MCF-7 TamR cells highlighted miR-181a-2 as one of the significantly overexpressed miRNAs present in MCF-7 TamR cells as well as cells derived exosomes associated with the ERα pathway [162]. Transfection of miR-181a-2 into MCF-7wt cells induced a transient transformation of these cells into tamoxifen-resistant cells. Additionally, treatment of MCF-7 sensitive cells with exosomes isolated from MCF-7 TamR cells led to the suppression of ERα and activation of Akt, ultimately resulting in tamoxifen resistance [162]. Moreover, Zhang et al. [169] demonstrated that androgen-dependent prostate cancer cells (ADPCs) can develop tolerance to androgen deprivation through exosome-mediated communication with androgen-independent prostate cancer cells (AIPCs) by upregulating heme oxygenase 1 (HMOX1). Notably, in castrated non-obese diabetic mice having SCID mutation (NOD/SCID) mice models, ADPCs alone were incapable of forming tumors. However, when ADPCs treated with exosomes from AIPCs were injected into castrated SCID mice, increased tumorigenicity was observed, indicating exosome-mediated androgen deprivation tolerance in ADPCs in vivo [169]. Furthermore, Li et al. [182] revealed that approximately 29 miRNAs were dysregulated in exosomes derived from paclitaxel-resistant prostate cancer cells compared with parental paclitaxel-sensitive prostate cancer cells. Analysis using DIANA-Tarbase v6.0 database for target prediction along with pathway analysis implicated these dysregulated miRNAs in targeting AR, PTEN, and T cell factors/lymphoid enhancer-binding factor 4 [182].

Numerous studies have documented the influence of exosomes on NRs in the context of cancer-related disruptions in the immune system and inflammation. Particularly, a paradigmatic example involving the interplay between sonic hedgehog subtype of medulloblastoma cells and tumor-associated macrophages (TAMs), elucidated the role of exosomes in tumor progression [167]. Medulloblastoma-derived exosomes were found to have reduced levels of let-7i-5p and miR-221-3p, prompting the induction of M2 polarization in TAMs through the upregulation of PPARγ. Intriguingly, enhanced anti-cancer activities were observed upon the administration of a PPARγ antagonist in conjunction with an inhibitor targeting sonic hedgehog signaling intermediate molecule, smoothened [167]. Additionally, another investigation unveiled the upregulation of miR-181a-5p in osteosarcoma tissues and exosomes derived from SAOS-2 osteosarcoma cells [168]. These exosomes were shown to downregulate ROR expression and induce M2 polarization of macrophages [168]. Furthermore, inhibition of miR-181a-5p in SOAS-2 derived exosomes reduced the activation of M2 macrophages, while knockdown of *ROR* in macrophages reversed the biological effects attributed to exosomal miR-181a-5p in macrophage activation [168].

Collectively, these investigations elucidate the phenomenon in which cancer cells harness exosomes as carriers to transmit cargo selectively targeting NRs, thereby entraining adjacent cells and immune entities. This orchestration ultimately leads to enhanced immune evasion, increased invasive tendencies, augmented migratory capacities, therapeutic resistance and facilitated metastasis. Indeed, studies have highlighted the reciprocal interaction, wherein immune cells deliver miRNAs targeting NRs to cancer cells via exosomal transport, inducing apoptosis and impeding the metastatic cascade. Consequently, there is an urgent need for meticulous and tailored explorations aimed at delineating specific interactions between exosomes and NRs in order to foster the development of innovative therapeutic modalities tailored to combat cancer cells.

### CVDs

CVDs, particularly coronary artery disease (CAD), continue to be the leading global cause of mortality [183–185]. Despite significant advancements in therapeutic strategies, including early revascularization after acute coronary syndromes, reduction of cholesterol level, and inhibition of the renin–angiotensin–aldosterone system, CVD remains a formidable health challenge [184]. As a result, concerted efforts have been made to investigate and identify novel risk factors for atherosclerosis that can be therapeutically targeted to improve primary and secondary prevention of CAD [184]. Importantly, recent studies have explored the potential involvement of NRs and the interplay of exosomal communication, suggesting that understanding and manipulating this interaction could yield therapeutic benefits for individuals with CVDs. For instance, Liu et al. [159] demonstrated that exosomes derived from the perivascular adipose tissue (PVAT) of atherosclerotic patients had lower levels of miR-382-5p in comparison to individuals without atherosclerosis. Notably, the inhibition of miR-382-5p in PVAT-derived exosomes resulted in a reduction in the formation of macrophage foam cells and increased cholesterol efflux. Mechanistic insights have revealed the involvement of the PPARγ/bone morphogenic protein 4 (BMP4) axis in the upregulation of cholesterol efflux, mediated through the modulation of adenosine triphosphate (ATP)-binding cassette A1 (ABCA1) and ATP-binding cassette G1 (ABCG1) [159]. Pretreatment with BMP4 abrogated PVAT induced upregulation of PPARγ, ABCA1 and ABCG1, further confirming the role of BMP4 in atherosclerosis [159]. Besides, exposure of HUVECs to exosomes derived from visceral fat carrying elevated levels of miR-27b-3p resulted in increased expression of proinflammatory genes, including *VCAM1*, *ICAM1*, and monocyte chemoattractant protein 1 (*MCP1*) [160]. A positive correlation has been observed between plasma exosomal miR-27b-3p and body mass index (BMI) or waist size in CAD patients. Mechanistically, miR-27b-3p was found to suppress PPARα by binding to the coding region of its mRNA and thereby activating NF-κB. Subsequent administration of a miR-27b-3p mimic induced inflammation and atherosclerosis in apolipoprotein E deficient (ApoE^−/−^) mice, whereas PPARα overexpression counteracted these effects and provided protection against atherosclerosis [160]. Findings from these studies suggest that exosomes exhibit the capacity to target recipient cells by exerting regulatory influence on NRs through the conveyance of miRNAs. This intricate interplay has a critical role in the pathological processes underlying the development and progression of CVDs.

### Chronic rhinosinusitis (CRS)

CRS is a persistent inflammatory condition affecting the paranasal sinuses, leading to a significant health burden due to its widespread occurrence and impact on patients’ quality of life [186, 187]. The primary therapeutic approach involves the administration of corticosteroids and anti-inflammatory agents delivered either nasally or orally. Nasal sprays are favored to mitigate potential systemic side effects [187]. Notably, a new class of medications called “biologic agents” has been approved for treating a specific subtype of chronic sinusitis characterized by the presence of polyps-grape-like swellings in the sinus lining [187]. Despite these advancements, achieving a lasting cure remains challenging as inflammation often recurs upon discontinuation of these medications [187]. Exosomes that modulate NRs have been shown to contribute to CRS-related inflammation, suggesting that druggable transcription factors such as NRs may have potential benefits in treating CRS. In a separate study, it was demonstrated that human nasal epithelial cells exposed to air particulate matter secrete and deliver exosomes enriched with miR-19a and miR-614 to macrophages, thereby exacerbating inflammation through upregulation of proinflammatory cytokines such as IL-1α, IL-1β, IL-6, and TNF-α [29]. This study also revealed that these exosomal miRNAs bind to the 3’UTR of RORα mRNA, suppressing the expression of RORα [29]. Moreover, increased expression of miR-19a and miR-614, coupled with decreased RORα levels, were observed in tissues from CRS patients compared to normal individuals. This indicates that exosome-mediated transport of miR-19a and miR-614 contributes to airborne allergic rhinosinusitis through the downregulation of RORα and may serve as potential therapeutic targets for treating CRS [29].

### HIV/acquired immune deficiency syndrome (AIDS)

HIV infection, commonly known as AIDS, is considered as one of the most formidable diseases of the twenty-first century, with profound social, financial, and political implications in both developed and developing nations [188]. As an immunological disorder, HIV weakens the immune system, leading to increased susceptibility to mortality from opportunistic comorbidities, including tuberculosis, septicemia, and pneumonia [188]. The morbidity and mortality associated with HIV type-1 (HIV-1) related diseases have markedly declined due to the introduction of potent antiretroviral therapy [189]. This therapeutic approach achieves sustained suppression of HIV-1 replication and gradual restoration of CD4^+^ T cell counts. Nevertheless, approximately 10 – 40% of individuals with HIV-1 infection do not attain normalization of CD4^+^ T cell counts despite sustained virological suppression [189]. Notably, immunological non-responders face an elevated risk of clinical progression to AIDS and non-AIDS events, displaying higher mortality rates compared to HIV-1 infected individuals with effective immune reconstitution [189]. Therefore, there is an urgent need to identify novel druggable targets aimed at improving the prognosis of these patients. Importantly, exosomes involved in the modulation of NRs have emerged as crucial contributors to AIDS progression. For example, Yuan et al. [172] demonstrated that macrophages infected with HIV-1 proteins Tat or gp-120 exhibit elevated expression levels of exosomal miR-23a and miR-27a. Exosomes enriched with miR-23a released from Tat-treated macrophages were found to exert an impact on the mitochondrial bioenergetics of recipient lung epithelial cells through the downregulation of PPARγ [172]. Hence, obstructing intercellular communication within the pulmonary microenvironment emerges as a prospective paradigm for pioneering interventional strategies aimed at immune modulation in the context of HIV.

### NASH

NASH affects approximately 40% of the global adult population and stands as a prominent contributor to end-stage liver diseases, including HCC and liver failure [190]. Despite the substantial medical need for addressing, halting, or reversing NASH, no approved drugs have been licensed so far, and the development of such therapeutics has proven to be challenging [191]. Research has unveiled the involvement of exosomes targeting NRs in the development and progression of NASH, potentially laying the foundation for future novel therapeutic development. Notably, a study demonstrated that exosomes derived from human umbilical cord mesenchymal cells (hUC-MSCs) alleviated NASH by modulating the expression of key factors such as sterol regulatory element-binding protein 1c, fatty acid binding protein 5, carnitine palmitoyl transferase 1α, acyl-CoA oxidase, fatty acid synthase, and PPARα [30]. Additionally, these exosomes exhibited anti-inflammatory effects by suppressing TNF-α and IL-6 associated with macrophages, while enhancing the phosphorylated-nuclear factor erythroid 2-related factor 2 (p-Nrf2)/Nrf2 ratio [30]. The involvement of this pathway may be crucial in developing new therapeutic protocols. Similarly, another study showed that intravenously administered hUC-MSC-derived exosomes in an MCD diet-induced NASH mouse model ameliorated liver inflammation, liver damage, and weight loss [174]. The administration of these exosomes was associated with the suppression of proinflammatory cytokines such as IL-1β, IL-6, and TNF-α, along with an increase in the anti-inflammatory cytokine IL-10, M2 macrophage markers such as arginase and CD206 levels, indicating an enhanced anti-inflammatory macrophage phenotype [174]. Furthermore, these exosomes reversed the downregulated levels of PPARα in oxidized LDL-treated hepatocytes, providing protection to hepatocytes against NASH [174]. Therefore, exosomes modulating PPARα present a promising therapeutic approach for NASH treatment.

### Neuronal inflammation and neurodegenerative diseases

The demographic shift towards an aging global population has resulted in neurological disorders accounting for 6.3% of the overall global disease burden [192, 193]. These disorders pose significant health challenges, leading to increased disability rates and demand for extended medical treatment [192]. Parkinson’s disease (PD), Huntington’s disease, and Alzheimer’s disease are the primary neurodegenerative diseases that exhibit symptoms, ranging from cognitive impairment to motor and respiratory difficulties [194]. Contributing factors include oxidative stress, neuroinflammation, mitochondrial dysfunction, protein misfolding, and aggregation, implicating these processes in the development and pathogenesis of neurological disorders [194, 195]. Extensive research efforts aim to unravel these complexities and identify potential therapeutic targets in the ongoing battle against neurological disorders. NRs and exosomes have shown significance in the pathology of these diseases. For instance, Ye et al. [158] unveiled an overexpression of exosomes containing miR-27-3p in individuals with acute cerebral injury (ACI). Upon administration of these exosomes into a rat model subjected to middle cerebral artery occlusion, inflammation worsened through targeting PPARγ. Therefore, the manipulation of the miR-27-3p/PPARγ axis may emerge as a prospective and innovative intervention for ameliorating ACI. Another study delved into the therapeutic potential of exosomes enriched with Wnt5 sourced from BMSCs in addressing sleep-related disorders in rat models of PD [176]. Treatment with these exosomes demonstrated a notable increase in dopamine and 5-hydroxytryptamine levels, accompanied by elevated PPARγ expression, enhanced sleeping time, reduced awaken time and restoration of mitochondrial membrane potential within the stratum of PD rats [176]. These results suggest that exosomes enriched with Wnt5 from BMSCs have the potential to rectify circadian rhythm-related abnormalities associated with PD, primarily by increasing the expression of PPARγ [176].

### Obesity, hypertension, and diabetes

The global rise in obesity since 1975 poses a significant contemporary healthcare challenge. Individuals with a BMI of 30.0–34.9 kg/m^2^ face a hazard ratio for overall mortality elevated by over 40%, reaching 100% at a BMI > 40 kg/m^2^, contributing to 4–9% of cancer diagnoses [196–198]. The development of effective anti-obesity medications encounters technical and societal challenges, with historical failures linked to adverse cardiovascular effects, elevated suicidal risk, and increased potential for drug dependence and abuse [196]. Hence, there exists a crucial imperative to establish enduring pharmacotherapy for achieving body weight normalization, accompanied by necessary tolerability and safety measures which has proven to be a formidable task [196]. An abundance of investigations has elucidated that exosome play a pivotal role in the progression of obesity and associated diseases through their regulation of NRs, implicating their involvement in molecular mechanisms of body weight regulation and potential as druggable agents. Notably, Castano et al. [175] demonstrated an upregulation of 4 miRNAs associated with obesity, namely miR-192, miR-122, miR-27α-3p, and miR-27b-3p, in exosomes. Administration of exosomes from obese mice to lean mice resulted in increased glucose intolerance and insulin resistance [175]. Similarly, lean mice treated with control exosomes transfected with obesity-associated miRNAs exhibited augmented central obesity and hepatic steatosis, indicating similar effects. Additionally, mice subjected to an HFD or those overexpressing miRNA mimics displayed increased triglyceride activation, and free fatty acids, diminished PPARα levels in eWAT and enhanced PPARα levels in liver tissues [175]. This was accompanied by an increase in glucose intolerance and hepatic inflammation. Notably, treatment with fenofibrate, a PPARα agonist, resulted in a reversal of these exosome induced effects [175]. Moreover, Zhang et al. [177] observed an elevation in plasma exosome concentrations enriched with miR-211 in rats with pulmonary hypertension. Injection of rats with miR-211 enriched exosomes exacerbated pulmonary hypertension, whereas inhibition of miR-211 led to its attenuation. Mechanistically, the overexpression of miR-211 enhanced the proliferation of pulmonary arterial smooth muscle cells by inhibiting calmodulin-dependent kinase 1 and PPARγ [177]. Further, Liu et al. [157] revealed that exosomes derived from macrophages in adipose tissue enriched with miR-29a induced insulin resistance in adipocytes, hepatocytes, and myocytes in preclinical settings. Furthermore, PPARδ was identified as a downstream target of miR-29a, and treatment with GW501516, a PPARδ agonist, partially mitigated insulin resistance induced by miR-29a [157]. Another study by Li et al. [171] demonstrated that exosomes derived from endothelial progenitor cells promoted proliferation and migration, and inhibited apoptosis of HaCaT cells under high glucose conditions. These exosomes showed wound-healing benefits in diabetic mice with skin injury. Exosomal miRNA profiling identified miR-182-5p as highly upregulated, with mechanistic insights revealing PPARγ as its direct target [171]. Taken together, these studies highlight the importance of exosomes derived from obese adipose tissues in modulating PPAR, contributing to the exacerbation of inflammation and complications associated with obesity. Therefore, targeting exosome-mediated modulation of PPAR may hold therapeutic potential in the context of obesity.

To summarize, these investigations revealed that exosomes derived from host cells intricately regulate various signaling pathways, encompassing cholesterol efflux and metabolism, EMT pathway, cytokine signaling, and estrogen-mediated carcinogenesis signaling. This regulatory influence significantly impacts the pathogenesis of chronic diseases. Importantly, these exosomes demonstrate a regulatory role in NRs such as PPARα, PPARγ, PPARδ, RORα, ERα, and ERβ, across a range of chronic diseases, including AIDS, atherosclerosis, cancer, CRS, diabetes, NASH, neurological diseases and obesity. The observed patterns suggest that manipulating exosomes targeting NRs represents an innovative and promising therapeutic strategy for the treatment of diverse chronic diseases.

## NRs targeting exosomes

**Fig. 5:**
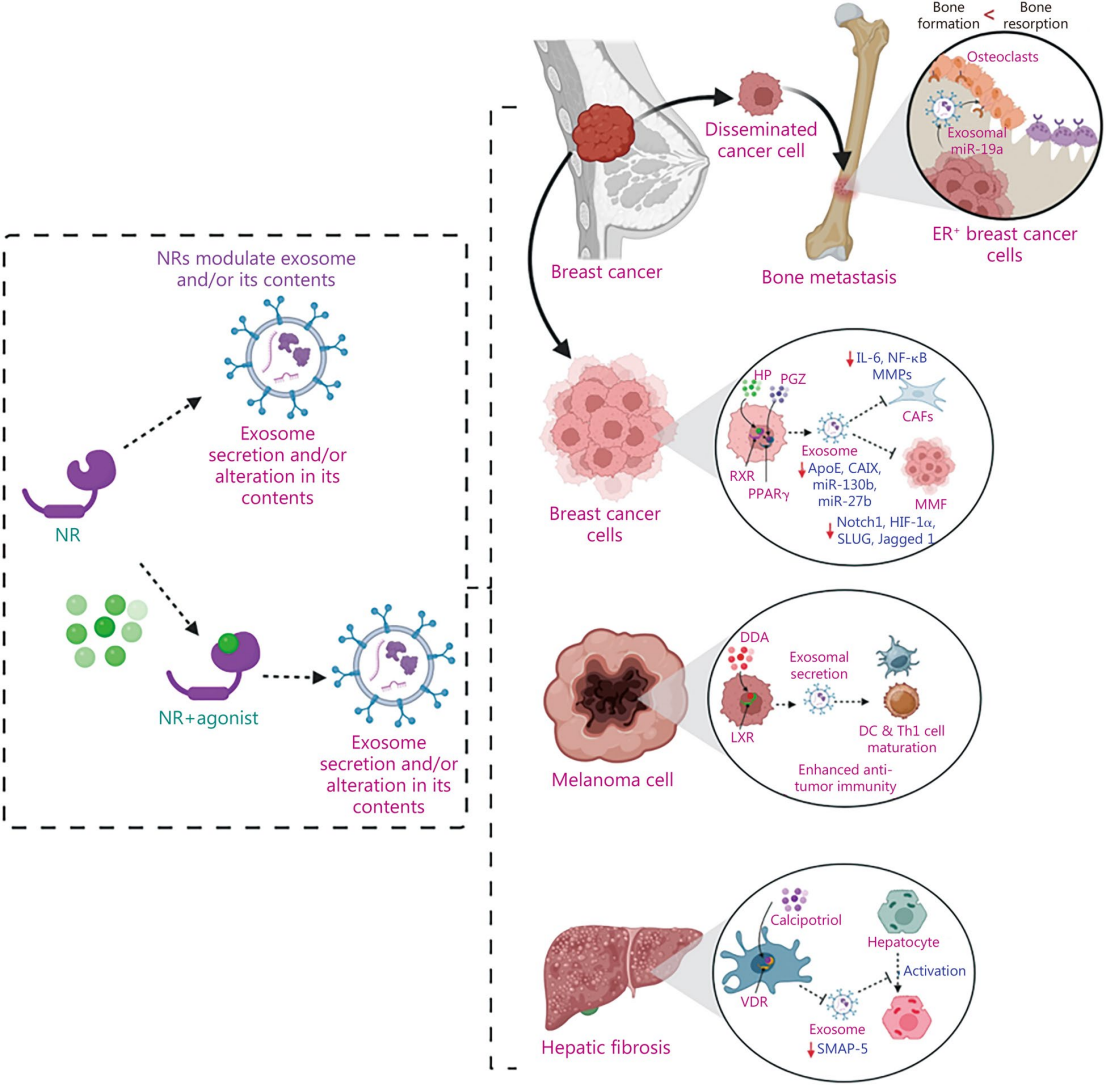
Nuclear receptors (NRs) regulate exosome secretion or alter its contents to modulate signaling pathways associate with the development and progression of various chronic diseases. Upregulation of exosomal miR-19a and integrin-binding sialoprotein in the tumor microenvironment has been shown to foster osteoclast attraction leading to metastatic seeding. RXR agonist 6-OH-11-O-hydroxyphenanthrene (HP) treatment potentiated pioglitazone (PGZ)’s inhibition of mammosphere formation in breast cells, reducing stem cell markers. Moreover, LXR ligand (DDA) inducing DDA-sEVs, promoting DC maturation, Th1 polarization, and inhibiting melanoma growth in mice. Further, VDR agonist calcipotriol was shown to reduce M2 polarization in macrophages, downregulating exosomal SMAP-5, causing greater reduction in hepatic fibrosis. ApoE apolipoprotein E, CAIX carbonic anhydrase IX, DC dendritic cells, DDA dendrogenin A, ER estrogen receptor, HIF-1α hypoxia-inducible factors-1α, IL interleukin, LXR liver X receptor, miR microRNA, MMF mammosphere formation, MMP matrix metalloproteinase, NF-κB nuclear factor kappa-B, PPAR peroxisome proliferator-activated receptor, RXR retinoid X receptor, SMAP-5 smooth muscle cell-associated protein protein-5, SLUG snail family transcriptional repressor 2, VDR vitamin D receptor

Recent studies have revealed the intricate involvement of NRs in targeting exosomes, thereby contributing to the initiation and progression of chronic diseases. The role of NRs in modulating exosomes and their contents during the development and progression of chronic diseases has been summarized in Table 2 and Fig. 5 [32, 33, 199, 200]. Notably, Wu et al. [199] demonstrated an upregulation of exosomal miR-19a and integrin-binding sialoprotein (IBSP) in ER^+^ breast cancer cells. Functionally, IBSP in these cells was shown to attract osteoclast cells, facilitating the transfer of exosomal miR-19a and creating a conducive tissue microenvironment for the colonization of breast cancer cells in bone [199]. In another study, treatment with 6-OH-11-O-hydroxyphenanthrene, an RXR agonist, potentiated the ability of pioglitazone, a PPARγ ligand, to impede mammosphere formation in MCF-7 breast cancer cells [32]. This effect was associated with a reduction in the expression of stem cell markers, including Notch1, Jagged 1, snail family transcriptional repressor 2 (Slug/SNAI2), HIF-1α, ApoE, IL-6, and carbonic anhydrase IX (CAIX) [32]. Intriguingly, these NR agonists counteracted the capacity of exosomes enriched with ApoE, CAIX, miR-130b, and miR-27b derived from hypoxic MCF-7 cells to induce a proinflammatory phenotype on breast fibroblasts [32]. Furthermore, these agonists were shown to reduce IL-6, NF-κB, matrix metalloproteinase (MMP)2, and MMP9 on tumor-associated fibroblasts under hypoxic conditions. Thus, the study indicated that NRs are involved in the inflammatory communication between cancer cells and fibroblasts, and manipulating this interaction may lead to the reprogramming of the tumor microenvironment [32]. Moreover, Record et al. [200] reported that treatment of SKMEL-28 melanoma cells with dendrogenin A (DDA), a ligand of LXR, resulted in the secretion of small extracellular vesicles (DDA-sEV) enriched in lipidated proteins and lipids. These DDA-sEVs induced DC maturation and Th1 polarization, ultimately inhibiting the growth of tumors in xenograft mice model [200]. This suggests the potential of targeting LXR as a novel strategy to enhance immunity against cancer cells through exosomes [200]. Modulation of exosomes by NRs has been established in hepatic fibrosis as well. For instance, Liu et al. [33] showed that exosomes derived from M2 macrophages can stimulate hepatic stellate cell activation; however, treatment of macrophages with calcipotriol, a VDR agonist, resulted in reduced M2 polarization and hepatocyte activation by downregulating exosomal smooth muscle cell-associated protein-5 (SMAP-5). Combining calcipotriol with a macrophage-targeted exosomal secretion inhibitor, GW4869 encapsulated in liposome (GWLP) exhibited stronger suppression of SMAP-5, hepatocyte activation, and enhanced repair of the liver structure. This combination therapy presents a promising approach against hepatic fibrosis [33].

**Table 2:** Molecular mechanism underlying nuclear receptors (NRs) that modulate exosomes secretion/contents in the pathogenesis of chronic diseases

Disease	NR	Exosomal content	In vitro/in vivo/clinical	Cell line/model	Intervention/Treatment/Expression modulation	Mechanism of action	References
Cancer							
Breast cancer	RXR/PPARγ	ApoE, CAIX, miR-130b & miR-27b	In vitro	MCF-MS	PGZ + IIF	↓MS, *Notch 3, Jagged, IL-6, Slug,* CAIX, HIF-1α	[32]
				TAF	PGZ + IIF	↓IL-6, IL-8, NF-κB promoter, MMP2, MMP9, tube formation, CD44	
					PGZ + IIF Exo	↓MS, *Notch 3*	
	ER	miR-19a & IBSP	In vitro	mBMM	MCF7BoM2-exo	↓PTEN ↑p-Akt, p-p65, OC proliferation & OC size	[199]
				MCF-7 BoM	–	↑miR-19a, IBSP	
				RAW264.7	miR19a	↑miR19a, size of osteoclast, p-p65, p-Akt ↓PTEN	
				mBMM	MCF7BoM2-miR19aKO-exo	↑PTEN ↓p-Akt, p-p65, OC proliferation, OC size	
			In vivo	Athymic nude mice	MCF7BoM2-IBSPKO-exo	↑Wound healing, bone density ↓Metastasis, osteoclastogenesis	
					MCF7/IBSP/miR-19a	↑Bone metastasis, bone density, osteoclastogenesis	
					T47D/IBSP/miR-19a	↑Tumor burden, bone metastasis bone density, osteoclastogenesis	
					MCF7BoM2/miR-19aKO	↓Bone metastasis osteoclastogenesis	
					Exo19aKO + IBSP	↑Bone density ↓Osteoclastogenesis	
					MCF7BoM2 + GW4869	↑Bone density ↓Bone metastasis free survival, osteoclastogenesis	
					T47DBoM2 + CGA	↓Metastasis, osteoclast ↑Bone density	
					MCF7BoM2 + CGA	↓Tumor volume	
			Clinical	ER^+^ breast cancer patients	–	↑Osteoclast number, osteoclast differentiation	
Melanoma	LXR	Lipidated proteins and lipids	In vitro	Th1	(DDA-Sev + shC) SKMEL-28	↑HLA-DR, CD86, CD54, CD80, IL-6, IL-12, CD40, CD83, TNF-α, IFN-γ, IL-5, IL-13	[200]
				B16F10	DDA	↑Exosome release, BMP, cholesterol protein & calreticulin in sEV	
				SKMEL-28	DDA-sEV	↓Cell viability ↑Cell cycle arrest	
					ShC + DDA	↑Tyr, TRP2, CD63, BMP ↓Rab27b	
					shLXR + DDA	↓Tyr, TRP2, CD63, BMP ↑Rab27b	
			In vivo	C57BL/6 (B16F10 xenografts)	DDA-sEV	↓Tumor volume	
Hepatic fibrosis	VDR	SMAP-5	In vitro	LX-2	M2-CM	↑Collagen-1, α-SMA, cell viability	[33]
					(M2 + calcipotriol)-CM	↓Collagen-1, α-SMA, cell viability	
					M2^VDR−KO^-CM	↑Collagen-1, α-SMA, cell activation	
					(M2 + GW4869)-CM	↓Cell activation, collagen-1, α-SMA	
					(M2 + calcipotriol) Exo	↓LC3A/B-II ↑p62	
					(M2 + siSMAP-5) exo	↓Collagen-1, α-SMA, SMAP-5, ATG5, ATG7, ATG12	
					(MO-SMAP-5-OE) exo	↑Collagen-1, α-SMA, SMAP-5, ATG5, ATG7, ATG12	
			In vivo	CCl4 induced fibrotic C57BL/6 mice	GWLP + calcipotriol	↓α-SMA, ALT, AST, TBA, collagen-1 ↑Repair of liver tissue	
					AAV-shSMAP-5	↓F4/80^+^ macrophages, serum ALT, AST, and TBA, α-SMA	

*α-SMA* α-smooth muscle actin, *ALT* alanine aminotransferase, *ApoE* apolipoprotein E, *AST* aspartate aminotransferase, *ATG* autophagy related, BMP bone morphogenetic protein 2, *CAIX* carbonic anhydrase IX, *CCl4* carbon tetrachloride, *CGA* chlorogenic acid, *DDA* dendrogenin A, *ER* estrogen receptor, *HIF-1α* hypoxia-inducible factors-1α, *HLA-DR* Human Leukocyte Antigen-DR isotype, *IBSP* integrin-binding sialoprotein, *IFN-γ* interferon-γ, *IIF* 6-OH-11-O-hydroxyphenanthrene, *IL* interleukin, *LXR* liver X receptor, *mBMM* mouse bone marrow monocyte, *miR* microRNA, *MMP* matrix metalloproteinase, *MS* mammospheres, *NF-κB* nuclear factor-kappa B, *OC* osteoclast, *PGZ* pioglitazone, *PPAR* peroxisome proliferator-activated receptor, *PTEN* phosphatase and tensin homolog, *Rab27b* member of RAS oncogene family, *RXR* retinoid X receptor, *SMAP-5* smooth muscle cell-associated protein*-*5, *Slug/SNAI2* snail family transcriptional repressor 2, *TAF* tumor associated fibroblast, *TBA* total bile acid, *TNF-α* tumor necrosis factor-α, *Tyr* tyrosinase, *TRP2* tyrosinase-related protein 2, *VDR* vitamin D receptor

In a concise summary, NRs intricately facilitate bidirectional communication between host and recipient cells within the tissue microenvironment by regulating either the process of exosomal secretion or the composition of exosomal cargo. Research has revealed the capacity of NRs to modulate the lipid and lipidated protein components within exosomes derived from cancer cells. Disruption in these NR-mediated pathways is associated with the onset and/or progression of chronic diseases. Therefore, we believe that a comprehensive exploration of these intercellular dialogues holds promise for the development of innovative therapeutic approaches targeting chronic diseases.

## Therapeutic application of targeting exosome-NR axis

Exosomes, containing miRNAs, lncRNAs, and proteins, represent a novel reservoir of biomarkers for various diseases [201–204]. Through mechanisms such as clathrin-mediated endocytosis, lipid-raft-mediated endocytosis, caveolin-mediated endocytosis, phagocytosis, and micropinocytosis, exosomes deliver their cargo into cells and binding to target sites [43]. This explains the diverse role of exosomes as a key regulator of cell-to-cell communication in both normal and pathological conditions [205]. The exosome-NR axis emerges as a promising therapeutic target for chronic diseases based on the abundance of miRNAs in exosomes that can modulate the expression of NRs by directly binding to their 3’UTRs or indirectly regulating their downstream pathways, leading to immune cell activation, apoptosis, variation in survival, proliferation, metastasis, and inducing metabolic rewiring such as alterations in mitochondrial and glucose metabolism [163, 165]. Exosomes have a significant role in disease progression by modulating PPAR. For instance, exosomes released by macrophages upon HIV infection exosomes were enriched with miR-27a and miR-23a that subsequently bind to PPARγ and ZO-1 respectively, further leading to altered mitochondrial function and compromised tight junction integrity in alveolar cells, thereby contributing to lung injury and infection  [172]. This finding may help to improve immune dysfunction during HIV infection by targeting exosomal content or PPARγ  [172]. Exosomes from rats with pulmonary hypertension exhibited increased expression of miR-211 and exacerbated the disease by downregulating calcium/calmodulin dependent protein kinase I and PPARγ expression in lung tissues [177]. Another study demonstrated that adipose MSCs aid in wound healing through lncRNA H19. H19 present in exosomes can target and inhibit miR-130b-3p and lead to the activation of downstream PPARγ and STAT3, ultimately resulting in the polarization of M2 macrophage, thereby enhancing cell proliferation, angiogenesis and inhibited inflammatory response in preclinical settings [31]. TAM-derived exosomes showed reduced levels of let-7i-5p and miR-221-3p, which are inversely correlated with PPARγ expression in medulloblastoma [167]. Enhanced expression of PPARγ promotes M2 macrophage polarization and tumor progression, but these effects were reversed by treatment with its antagonist [167]. Furthermore, a study has demonstrated the immunosuppressive role of PPARα in cancer, with TDEs containing long-chain fatty acids, increasing lipid content in DCs via PPARα activation, suggesting the potential for DC-based cancer therapy [163]. In another study, Liu et al. [173] elucidated the role of hepatocyte-derived exosomal miR-122-5p in mediating liver ischemia-reperfusion injury, particularly through the M1 polarization of Kupffer cells, involving the modulation of the PPARδ and NF-κB pathway in preclinical settings. Importantly, inhibition of exosomal miR-122-5p was associated with the suppression of M1 polarization in Kupffer cells, leading to the amelioration of liver ischemia-reperfusion injury [173]. Additionally, in obesity-induced insulin resistance, exosomes derived from adipose tissue macrophages exhibited significant upregulation of miR-29a, which can bind to PPARδ and cause insulin resistance [157]. Notably, treatment with a PPARδ agonist reversed this effect, demonstrating its potential as a therapeutic target against obesity-associated type II diabetes [157].

Previously study has also shown that ROR plays a vital role in the communication between exosomes and NRs [29]. Specifically, exposure to air pollution triggers the release of exosomes enriched with miR-19a and miR-614 from human nasal epithelial cells. These miRNAs have the potential to target RORα and thereby inhibit the transcriptional repression of proinflammatory gene expression  [29]. This subsequently led to the polarization of M1 macrophages and activation of inflammatory responses in a mucosal microenvironment [29]. Moreover, in glioma, hypoxia-induced upregulation of miR-10a and miR-21 resulted in the activation of MDSCs through RORα and PTEN respectively. This finding also sheds light on how hypoxia modulates the immune microenvironment through exosomes-mediated NR interactions [164].

Exosomes play a crucial role in mediating the transfer of chemoresistance, especially in cancers associated with steroid receptors, thereby contributing to increased tumor aggressiveness [161]. Exosomes from ER^+^ tamoxifen-resistant breast cancer cells exhibited smaller size and elevated levels of miR-221/-222 compared to exosomes from tamoxifen-sensitive cells. These miRNAs caused downregulation of ER and p27 in recipient cells, leading to increased survival and proliferation [161]. Similarly, another study showed that ADPCs such as LNCaP exhibits increased survival and proliferation after treatment with exosomes derived from AIPCs like PC3 [169]. Interestingly, after castration, LNCaP failed to form tumors, whereas treatment with PC3-derived exosomes resulted in tumor formation in prostate cancer along with increased expression of HMOX1. This upregulated expression might be associated with androgen resistance and activation of cancer-related pathways, leading to prolonged survival and proliferation of prostate cancer cells under androgen-deprived conditions [169]. This evidence suggests that the contents of exosomes are crucial for chemoresistance in steroid-related cancer cells. Therefore, targeting this pathway will be beneficial for treating chemoresistance in cancer cells.

Furthermore, studies have provided evidence that treatment with NR agonists induces the release of exosomes, thereby modulating metabolic pathways and resulting in reduced disease progression [32, 33]. For example, the combined administration of PPAR and RXR agonists inhibited the production of proinflammatory cytokines through exosome communication, leading to tumor suppression in breast cancer [32]. Similarly, the use of VDR agonists improved hepatic fibrosis by inhibiting the release of exosomes derived from macrophages [33]. Therefore, the administration of NR agonists holds promise in impeding tumor progression by regulating exosome release. Overall, these findings highlight exosome-NR interactions as a promising strategy for developing safe and effective therapeutic treatments.

## Safety and pharmacokinetics of exosomes

The initial concept of exosomes as cellular waste bins responsible for the disposal of surplus proteins, peptides, and nucleic acids has evolved with recent investigations revealing their potential as efficient drug delivery vehicles [206]. Nevertheless, a comprehensive consideration of exosome biodistribution, pharmacokinetics, and safety profiles is imperative for their application as drug delivery agents. In sepsis induced mice, systemically administered exosomes primarily distribute to major tissues including the liver, spleen, and lung [207]. The distribution patterns are influenced by factors such as the cellular origin of exosomes, membrane composition, and the host’s pathophysiological status [207].

Presently, bioluminescence and fluorescence imaging are the primary methods for characterizing exosome in vivo [208]. Exosomes labeled with gLuc-lactadherin, derived from B16-BL6 cells, were intravenously injected into a mouse model, revealing rapid clearance by the liver from the bloodstream [209]. Importantly, this investigation demonstrated the persistence of the gLuc-lactadherin label following intravenous administration, suggesting its potential utility in tracking exosome tissue distribution [209]. Another study examined the biodistribution and pharmacokinetics of exosomes derived from HEK293T cells in both sepsis and healthy mice, revealing an 80% clearance rate in healthy mice 1 h post-intravenous injection [207]. Similarly, therapeutic exosomes labeled with zirconium-89 (^89^Zr) were administered intravenously to mice and rat models, with lower ^89^Zr retention observed in rats compared to mice. Additionally, rapid exosome clearance from the bloodstream indicated prompt tissue absorption predominantly by the liver, with lesser uptake observed in the spleen and other organs [210].

Compelling evidence suggests that MSC-derived exosomes showed a superior safety profile compared to their host cells, as they are easily stored without losing functionality [211]. Moreover, a safety study has been conducted using exosomes derived from human induced pluripotent stem cells in in vivo models [212]. Tail vein injection and nasal administration of these  exosomes showed mild immune cell activation with no obvious negative trend [212]. Similarly, administration of exosomes derived from human umbilical mesenchymal stromal cells to rat models showed positive effects on weight reduction, with no apparent adverse effects observed on liver and renal function [213]. Additionally, in an acute lung injury model, the administration of exosomes from adipose-derived MSCs showed a potent protective effect by compensating the damaged mitochondria of macrophages [214]. This further transformed macrophages into an anti-inflammatory phenotype and reduced the oxidative stress in the mice model [214]. Exosomes have the capability to deliver therapeutic agents in a site-specific manner. Parolini et al. [215] demonstrated the tendency of exosomes to release their content at low pH value, which is a hallmark of cancer cells. Similarly, doxorubicin-entrapped exosomes administered to HER2^+^ TUBO cells exhibited increased binding affinity compared to HER2^−^ 4T1 cells [216]. Similar results were obtained from in vivo breast cancer mouse models as well [216]. These studies highlight the potential for utilizing exosomes as a safe and promising tool for targeted drug delivery in cancer treatment.

Several clinical trials have confirmed the safety and efficacy of exosomes in humans. For instance, a phase I/II clinical trial investigated the safety of allogenic human adipose MSC-exosomes in patients with mild to moderate Alzheimer’s diseases. The study demonstrated that human adipose MSC-exosomes exhibited no adverse effects along with reduced Alzheimer’s Disease Assessment Scale-Cognitive section (ADAS-cog) scores and improved cognitive function [217]. Moreover, administration of gel based adipocyte tissue stem cell-derived exosomes as post-treatment to fractional CO_2_ laser for acne scar in 25 patients showed a protective effect compared to the control group [218]. Similarly, positive outcomes were observed in a cohort of 60 patients undergoing treatment for melasma, wherein hUC-MSCs-exosomes were combined with microneedles, non-ablative fractional laser, or Peninsula Blue Aurora Shumin Master plasma. These results were attributed to the remarkable deep-penetrating capability, safety and efficacy of hUC-MSCs-exosomes in melasma treatment [219]. Another pilot study involving 7 COVID-19 pneumonia patients, evaluated the safety of MSC-derived exosomes. Interestingly, the results demonstrated that the exosomes did not cause allergic symptoms in patients and led to reduced hospitalization duration in mild COVID-19 cases [220]. Likewise, an open-label phase-IIa clinical trial involving severe COVID-19 patients reported no adverse effects following nubilization  with human adipose-derived mesenchyma stromal exosomes [221]. The safety profile of placental MSC-derived exosomes was established in a phase I clinical trial involving 11 patients with complex perianal fistulae, characterized by persistent fistulas for at least 1 year despite medical and surgical interventions [222]. Notably, complete resolution of fistula tracts was observed in 5 patients. None of the patients showed any acute or latent allergic reaction or injection related complications [222]. Similarly, in another investigation, treatment with MSC-derived exosomes resulted in complete healing of refractory perianal fistula in 3 out of 5 inflammatory bowel disease patients without  any systemic or local adverse events [223].

In conclusion, these studies confirm the safety and pharmacokinetics of exosomes in both clinical and preclinical settings. Further studies are imperative to elucidate their role in precision medicine and therapeutic interventions.

## Exosome engineering

Recently, cell-derived exosomes have gained increased attention as an advanced drug delivery system due to their low immunogenicity, high physicochemical stability, capacity to penetrate tissues, and long-distance communication abilities [224]. Accumulating evidence has elucidated various strategies for modifying exosomes to optimize their utility as drug delivery vehicles. These strategies include incubating drugs with exosomes and exosome-secreting donor cells, transfection, and employing physical methods such as extrusion, sonication, freeze–thaw cycles, and electroporation [225, 226]. For instance, Saari et al. [227] investigated the efficacy of prostate cancer-derived EVs, including exosomes and microvesicles, when incubated with paclitaxel in the treatment of prostate cancer. This study revealed that EVs conjugated with paclitaxel were endocytosed and exhibited potent cytotoxic effects. Interestingly, removal of surface receptors from microvesicles resulted in reduced cytotoxic effects, whereas no changes were observed in exosomes with the drug [227].

Additionally, research has shown the potential of macrophage-derived exosomes in treating central nervous system-associated disorders. Evidence suggests their ability to cross the blood–brain barrier, making them promising tools for delivering drugs to treat central nervous system diseases [228]. Kim et al. [229] studied the impact of macrophage-derived exosomes conjugated with paclitaxel on drug-resistant cancer cells. The incorporation of paclitaxel into exosomes using sonication methods resulted in high drug loading efficacy and enhanced cytotoxic effects on cancer cells. Furthermore, administering these exosomes through airway delivery demonstrated anti-cancer effects in a mouse model with pulmonary metastases of Lewis lung carcinoma [229].

Moreover, numerous studies have explored the potential of exosomes as a vehicle for drug delivery through exosome engineering. For example, the fusion of rabies virus glycoprotein with Lamp-2b protein expressed in the exosome membrane serves as a cell-penetrating peptide, facilitating the targeted delivery of exosomes containing siRNAs to the brain [230]. Rabies virus glycoprotein specifically binds to acetylcholine receptors present in neuro-endothelial and neuronal cells [230]. Additionally, new approaches are emerging in exosome therapy, such as exosomes for protein loading via optically reversible protein–protein interactions (EXPLORs) [231]. In this technique, cargo proteins are fused with cryptochrome circadian regulator 2 (CRY2) protein isolated from *Arabidopsis thaliana*, while truncated CRY-interacting basic-helix-loop-helix 1 is conjugated with CD9 protein, an exosome marker [231]. Upon blue light irradiation, the cargo protein fused with CRY2 undergo reversible interactions with CRY-interacting basic-helix-loop-helix 1, enabling entry into the inner surface of the cell membrane and loaded into exosomes following induction of exosome biogenesis. Subsequently the cargo can be released into to the exosome from the protein conjugated through the removal of blue light illumination. Notably, EXPLORs have shown superior efficiency compared to other methods of isolating exosomes [231]. Similarly, another genetically engineered exosome device known as EXOsomal transfer into cells (EXOtic), which contains a mRNA packaging device and cytosolic delivery helper, has shown potential therapeutic efficacy in preclinical studies by facilitating cargo delivery into brain cells as a promising treatment option for PD [232]. Another study evaluated biomimetic exosomes encapsulating dexamethasone sodium phosphate nanoparticles (Exo/Dex), whose surface was engineered with a folic acid-polyethylene glycol-cholesterol compound as a targeted drug delivery system for the treatment of rheumatoid arthritis. Interestingly, these exosomes exhibited no apparent hepatotoxic effects while demonstrating favorable biocompatibility [233].

Accumulating evidence from preclinical studies has highlighted the potential of engineered exosomes to selectively target different pathways, notably including NRs, as a promising therapeutic strategy against chronic diseases. As previously discussed, NRs regulate myriads of physiological and pathological conditions in the body through intricate downstream signaling pathways. Hence, utilizing engineered exosomes as delivery vehicles for NR modulators may represent a paradigm shift in the therapeutic approaches against various disorders.

## Challenges of targeting exosomes for therapy

The aforementioned investigations highlight the potential of exosomes as effective drug delivery vehicles for treating chronic diseases. However, there are significant obstacles that hinder their efficacy as drug delivery tools in both clinical and preclinical settings. These challenges include issues related to isolation, characterization, insufficient targeting capabilities, quality control, and limited reproducibility in preclinical models [224, 234]. The primary challenge associated with exosomes is their isolation. Exosomes are often found alongside other EVs, leading to heterogeneity that diminishes the therapeutic targeting efficacy. The traditional isolation method involving multistep ultracentrifugation is laborious process with a high risk of impurities [235]. Additionally, characterization is another crucial aspect, where exosomes isolated from the same cells show inconsistent properties that can affect the therapeutic efficacy [235]. Notably, exosomes can be considered as double-edged sword because they have the potential to either support or weaken health depending on context [236]. The cell uses exosomes to eliminate unwanted toxic compounds, thereby manitain donor cells homeostasis [237]. However, exosomes derived from cancer cells may contain oncogenic precursors and undesired cargo that can lead to harmful effects in the recipient system [234, 238]. Moreover, exosomes have a limited lifespan of approximately 2 h in the bloodstream and predominantly  cleared by macrophages. Besides, their poor zeta potential reduces efficacy by promoting aggregates, which can trigger an immune response and hinder their delivery to the target site [239]. Clinically, challenges related to stability, preservation, transportation, and cost constrain the use of exosomes [239]. The preservation temperature for exosomes varies depending on the patient’s tissue and must be set at either 4 °C or −80 °C, impacting their protein content [240]. Factors such as storage pH, buffering conditions, and freeze–thaw cycles, also affect the exosomal protein content [240]. Determining the appropriate dosage is a major challenge due to potential immune responses from incorrect dosing. Furthermore, the clinical application of exosome as a personalized medicine is limited by cost constraints. Therefore, there is a necessity for cost-effective and time-efficient nano techniques to develop exosome therapeutics that are both affordable and efficient [239].

Despite the challenges, research on exosomes is steadily progressing and approaching a new frontier. Several clinical trials are currently underway to explore the potential of exosomes as a therapeutic option. However, sustained research efforts in this field are crucial for addressing and overcoming the existing obstacles.

## Conclusion and future perspective

Chronic diseases continue to be a significant factor contributing to widespread morbidity and economic burdens, resulting in millions of fatalities globally. The advancement of modern medical technologies has led to the discovery of innovative therapeutic approaches, significantly improving both the quality of life and survival rates for affected individuals. However, these treatment methods often lead to adverse side effects and yield suboptimal clinical outcomes in the advanced stages of the diseases. The ongoing interest in cellular communication continually engages the scientific community with the overarching objective of discerning novel therapeutic modalities for addressing chronic diseases through cellular communication. Exosomes have recently gained considerable attention due to their pivotal role in cellular communication via both paracrine and endocrine signaling pathways. Additionally, NRs, as ligand-activated transcription factors, play a central role in maintaining bodily homeostasis by regulating relevant genes. Numerous studies have highlighted the significance of exosome-NR communication in various physiological and pathological contexts. This comprehensive review represents the first in-depth analysis integrating data on the interplay between NRs and exosomes, elucidating their implications in the initiation and progression of chronic diseases. The anticipated outcome of this novel cellular intercommunication is poised to offer a robust platform for the development of innovative therapeutic regimens. The emerging understanding of the interrelationship between NRs and exosomes highlights a contemporary avenue in cellular communication. Although the existing literature on this subject is limited, focused exploration of these interactions presents a prospective avenue for future scientific inquiry. Subsequent investigations are deemed essential to unravel the intricate molecular mechanism that underlies this phenomenon and discern its implications in both physiological and pathological contexts. This imperative seeks to expand our comprehension of the intricate interplay between NRs and exosomes, thereby fostering advancements in the field of cell biology and molecular signaling.

Interestingly, exosomes emerge as potential carriers for delivering biological molecules, such as miRNAs, with precise targeting capabilities based on their size, composition, and targeting precision to minimize adverse effects. The growing interest in utilizing exosomes as a therapeutic approach has attracted global research attention. Simultaneously, NRs have emerged as targets for developing novel therapeutic strategies. Metabolic activities of diseased cells undergo regulation through NRs, facilitated by exosomal miRNAs or siRNAs. Consequently, modulating NRs, exosomal contents, or both presents a promising avenue for novel treatment of chronic disease. However, existing studies remain insufficient, necessitating further studies. Crucially, elucidating the intricate mechanisms governing the reciprocal regulation between exosomal contents and NRs requires in-depth exploration. Furthermore, the limited number of clinical studies highlights the imperative for additional trials to deepen our understanding of the involvement of NRs in chronic diseases.

## Data Availability

Not applicable.

## Abbreviations

ABCA1: ATP-binding cassette A1
ABCG1: ATP-binding cassette G1
ACI: Acute cerebral injury
ADPCs: Androgen-dependent prostate cancer cells
AIDS: Acquired immune deficiency syndrome
AIPC: Androgen-independent prostate cancer cells
Akt: Protein kinase B
AP1: Activator protein 1
ApoE: Apolipoprotein E
APL: Acute promyelocytic leukemia
AR: Androgen receptor
ATRA: All-trans-retinoic acid
BMI: Body mass index
BMP4: Bone morphogenic protein 4
BMSCs: Bone marrow mesenchymal stem cells
CAD: Coronary artery disease
CAR: Constitutive androstane receptor
CRS: Chronic rhinosinusitis
CRY2: Cryptochrome circadian regulator 2
CVDs: Cardiovascular diseases
DAX1: Dosage-sensitive sex-reversal adrenal hypoplasia congenital critical region on the X chromosome gene 1
DC: Dendritic cell
DDA: Dendrogenin A
DNA: Deoxyribonucleic acid
DNMT1: DNA methyltransferase 1
EMT: Epithelial-mesenchymal transition
ER: Estrogen receptor
ERK: Extracellular signal-regulated kinase
ERR: Estrogen-related receptor
EVs: Extracellular vesicles
eWAT: Epididymal white adipose tissue
FXR: Farnesoid X receptor
GWLP: GW4869 encapsulated in liposome
HCC: Hepatocellular carcinoma
HER2: Human epidermal growth factor receptor 2
HFD: High-fat diet
h-GDEs: Hypoxic glioma-derived exosomes
HNF: Hepatocyte nuclear factor
HIV: Human immunodeficiency virus
HIV-1: Human immunodeficiency virus type-1
HIF: Hypoxia-inducible factor
HMOX1: Heme oxygenase 1
HNSCC: Head and neck squamous cell carcinoma
HPV: Human papillomavirus
hUC-MSCs: Human umbilical cord mesenchymal cells
HUVECs: Human umbilical vein endothelial cells
IBSP: Integrin-binding sialoprotein
ICAM1: Intercellular adhesion molecule 1
IL: Interleukin
JNKs: C-Jun NH2-terminal kinase
LDL: Low-density lipoprotein
lncRNA: Long non-coding RNA
LXR: Liver X receptor
LRH1: Liver receptor homologue 1
MAPK: Mitogen activated protein kinase
MCD: Methionine-choline deficient
MDSC: Myeloid-derived suppressor cell
miRNA: MicroRNA
MMP: Matrix metalloproteinase
MR: Mineralocorticoid receptor
MSCs: Mesenchymal stem cells
NAFLD: Non-alcoholic fatty liver disease
NASH: Non-alcoholic steatohepatitis
NF-κB: Nuclear factor kappa-B
NOD/SCID mice: Non-obese diabetic mice having SCID mutation
NQO1: NAD(P)H quinone dehydrogenase 1
NRs: Nuclear receptors
OC: Osteoclast
PAI-1: Plasminogen activator inhibitor-1
PD: Parkinson’s disease
PGC-1α: PPARγ coactivator-1 alpha
PHLPP: PH domain and leucine rich repeat protein phosphatases
PKA: Protein kinase A
PKC: Protein kinase C
PLP2: Proteolipid protein 2
PR: Progesterone receptor
PPAR: Peroxisome proliferator-activated receptor
PTEN: Phosphatase and tensin homolog
PVAT: Perivascular adipose tissue
PXR: Pregnane X receptor
RAR: Retinoic acid receptor
ROR: RAR-related orphan receptor
ROS: Reactive oxygen species
RXR: Retinoid X receptor
SA: Stable plaque atherosclerosis
SIRT: Sirtuin
SMAP-5: Smooth muscle cell-associated protein-5
STATs: Signal transducers and activators of transcription
TAM: Tumor-associated macrophage
TDEs: Tumor-derived exosomes
TGF-β: Transforming growth factor-β
TGR5: G protein-coupled receptor 5
THR: Thyroid hormone receptor
TME: Tumor microenvironment
TNF-α: Tumor necrosis factor-α
TR: Testicular receptor
UCEC: Uterine corpus endometrial cancer
UA: Unstable plaque atherosclerosis
VCAM1: Vascular cell adhesion molecule 1
VDR: Vitamin D receptor
